# Temporospatial inhibition of Erk signaling is required for lymphatic valve formation

**DOI:** 10.1038/s41392-023-01571-9

**Published:** 2023-09-11

**Authors:** Yaping Meng, Tong Lv, Junfeng Zhang, Weimin Shen, Lifang Li, Yaqi Li, Xin Liu, Xing Lei, Xuguang Lin, Hanfang Xu, Anming Meng, Shunji Jia

**Affiliations:** 1 State Key Laboratory of Membrane Biology, Tsinghua-Peking Center for Life Sciences, School of Life Sciences, Tsinghua University, Beijing, 100084 China; 2 Guangzhou Laboratory, Guangzhou, 510320 Guangdong Province China; 3 State Key Laboratory of Molecular Developmental Biology, Institute of Genetics and Developmental Biology, Chinese Academy of Sciences, Beijing, 100101 China

**Keywords:** Lymphangiogenesis, Differentiation

## Abstract

Intraluminal lymphatic valves (LVs) and lymphovenous valves (LVVs) are critical to ensure the unidirectional flow of lymphatic fluid. Morphological abnormalities in these valves always cause lymph or blood reflux, and result in lymphedema. However, the underlying molecular mechanism of valve development remains poorly understood. We here report the implication of Efnb2-Ephb4-Rasa1 regulated Erk signaling axis in lymphatic valve development with identification of two new valve structures. Dynamic monitoring of phospho-Erk activity indicated that Erk signaling is spatiotemporally inhibited in some lymphatic endothelial cells (LECs) during the valve cell specification. Inhibition of Erk signaling via simultaneous depletion of zygotic *erk1* and *erk2* or treatment with MEK inhibitor selumetinib causes lymphatic vessel hypoplasia and lymphatic valve hyperplasia, suggesting opposite roles of Erk signaling during these two processes. *ephb4b* mutants, *efnb2a;efnb2b* or *rasa1a;rasa1b* double mutants all have defective LVs and LVVs and exhibit blood reflux into lymphatic vessels with an edema phenotype. Importantly, the valve defects in *ephb4b* or *rasa1a;rasa1b* mutants are mitigated with high-level *gata2* expression in the presence of MEK inhibitors. Therefore, Efnb2-Ephb4 signaling acts to suppress Erk activation in valve-forming cells to promote valve specification upstream of Rasa1. Not only do our findings reveal a molecular mechanism of lymphatic valve formation, but also provide a basis for the treatment of lymphatic disorders.

## Introduction

Lymphatic vessels are components of the lymphatic system and play essential roles not only in immune cell trafficking, tissue fluid homeostasis, and lipid absorption, but also in a variety of pathological conditions, such as cancer progression and metastasis, lymphedema, immune responses, obesity, cardiovascular pathologies, glaucoma, and neurological diseases.^1–5^ Lymphatic development begins with the budding of lymphatic endothelial cell (LEC) progenitors that express Prox1 from the cardinal veins and other LEC sources.^3,6–8^ The expression levels of the essential transcription factors for valve development, including GATA2, FOXC2, and PROX1, are elevated in valve-forming LECs following the initiation of lymphatic fluid flow.^2,9,10^ Lymphatic vessels have one-way valves, including intraluminal lymphatic valves (LVs) and lymphovenous valves (LVVs), to ensure the unidirectional flow of lymphatic fluid. Invariably, morphological abnormalities in these valves impede normal fluid homeostasis and result in lymphedema.^10–12^ By revealing a bicuspid valve structure similar to that found in mammals, a recent study provided compelling evidence for the existence of LVs and LVVs in zebrafish facial lymphatic vessels (FLVs).^13,14^ However, it is unknown whether other valve structures exist in the lymphatic system of zebrafish.

Mitogen activated protein kinases (MAPKs), including ERK, JNK, and p38, perform critical roles in cell growth, migration, proliferation, differentiation, and apoptosis.^15–17^ The MAPK/Erk pathway is primarily initiated by GTP-bound Ras activating Raf. Raf phosphorylates Mek, which in turn phosphorylates Erks, the main components of the Ras-Raf-Mek-Erk signaling cascade.^18,19^ It has been demonstrated that Vegfc-Vegfr3-mediated stimulation of Erk signaling is the primary signaling axis maintaining *Prox1* expression in LEC progenitors and, therefore, promoting LEC proliferation and cell fate specification.^20–22^ Numerous studies have shown that the Erk pathway is vital to lymphatic development. Zebrafish embryos lacking Vegfr3 fail to initiate lymphatic vessel sprouting or differentiation.^23^ Transgenic expression of RAF1^S259A^, a gain-of-function RAF1 mutant associated with human Noonan syndrome, in the endothelial cells of mouse embryos activates Erk, leading to enhanced commitment of venous ECs to the lymphatic fate and eventual lymphangiectasia.^24^ Overexpression of Ras in the endothelial cell lineage of mice also causes lymphatic vessel hyperplasia.^25^ Additionally, treatment with MEK inhibitors alleviates lymphatic anomaly-associated clinical symptoms in a patient carrying an ARAF^S214P^ gain-of-function mutation and prevents increased LEC sprouting in human primary dermal lymphatic endothelial cells (HDLECs) or zebrafish transgenic larvae that overexpress ARAF^S214P,^.^26^ Although the involvement of Erk signaling in the formation of lymphatic vessels is well understood, less is known about its function in the generation of lymphatic valves. One report suggested that termination of Vegfr3 signaling by Epsin1/2 in collecting lymphatic trunks is required for normal LV development in mice, but the underlying mechanism has not been investigated.

Ephrin-Eph signaling mainly functions in attractive or repulsive processes, particularly in tissue boundary formation, axonal guidance, angiogenesis, and lymphangiogenesis, via directing cell adhesion, migration, and repulsion.^27–30^ The membrane-bound ligand Efnb2 and its tyrosine kinase receptor Ephb4 have been reported to be essential for vascular arterial-venous specification, angiogenic remodeling, and embryonic survival in mice.^31–33^ Ras p21 protein activator 1 (RASA1), a negative regulator of Ras via its GTPase activating protein (GAP) activity, was first discovered as a regulator of mouse embryonic growth, blood vessel formation, and neural tissue development.^34^ Human mutations in the EFNB2-EPHB4-RASA1 cassette are associated with both vascular and lymphatic diseases, including capillary malformation-arteriovenous malformation (CM-AVM), vein of Galen malformation (VOGM), and central conducting lymphatic anomaly (CCLA).^35–40^ A recent study suggests that Efnb2-Ephb4 influences lymphatic endothelial cell junction integrity by regulating cytoskeletal contraction.^41^ In addition, it has been revealed that Efnb2-Ephb4 forward signaling may promote valve maturation and survival during late embryonic and early postnatal development in mice.^36,42–44^ The mouse model with *Rasa1* deficiency demonstrates a requirement of *Rasa1* for continued LV and LVV development after initial specification.^45–48^ However, the regulatory relationship between Efnb2-Ephb4-Rasa1 and Erk signaling in valve development remains unclear.

Here, we find two more types of valve structures in the zebrafish, helping to gain a deeper understanding of the lymphatic system in this vertebrate animal model. Significantly, we disclose a specific regulation of Erk signaling during lymphatic valve development. Although Erk signaling is involved in LEC development, we show that Erk inhibition is necessary for zebrafish valve cell development. Erk activity must be repressed in the FCLV to initiate the valve-forming cell program. Then, further inhibition of Erk signaling by Efnb2-Ephb4 signaling upstream of Rasa1 appears essential for the specification of valve-forming cells. In addition, the dynamic expression pattern of Efnb2 in valve-forming LECs at the vessel bifurcation likely accounts for the restricted repression of Erk activity.

## Results

### Identification of new valves in the zebrafish lymphatic system

Although intact lymphatic vessels were imaged in the zebrafish some time ago,^49–52^ two pairs of bicuspid valves, including one pair of lymphatic valves (LVs) and another pair of lymphovenous valves (LVVs), have only been found in zebrafish facial lymphatic vessels (FLVs) more recently.^13^ However, whether there exist other specialized valve structures is unknown.

**Fig. 1:**
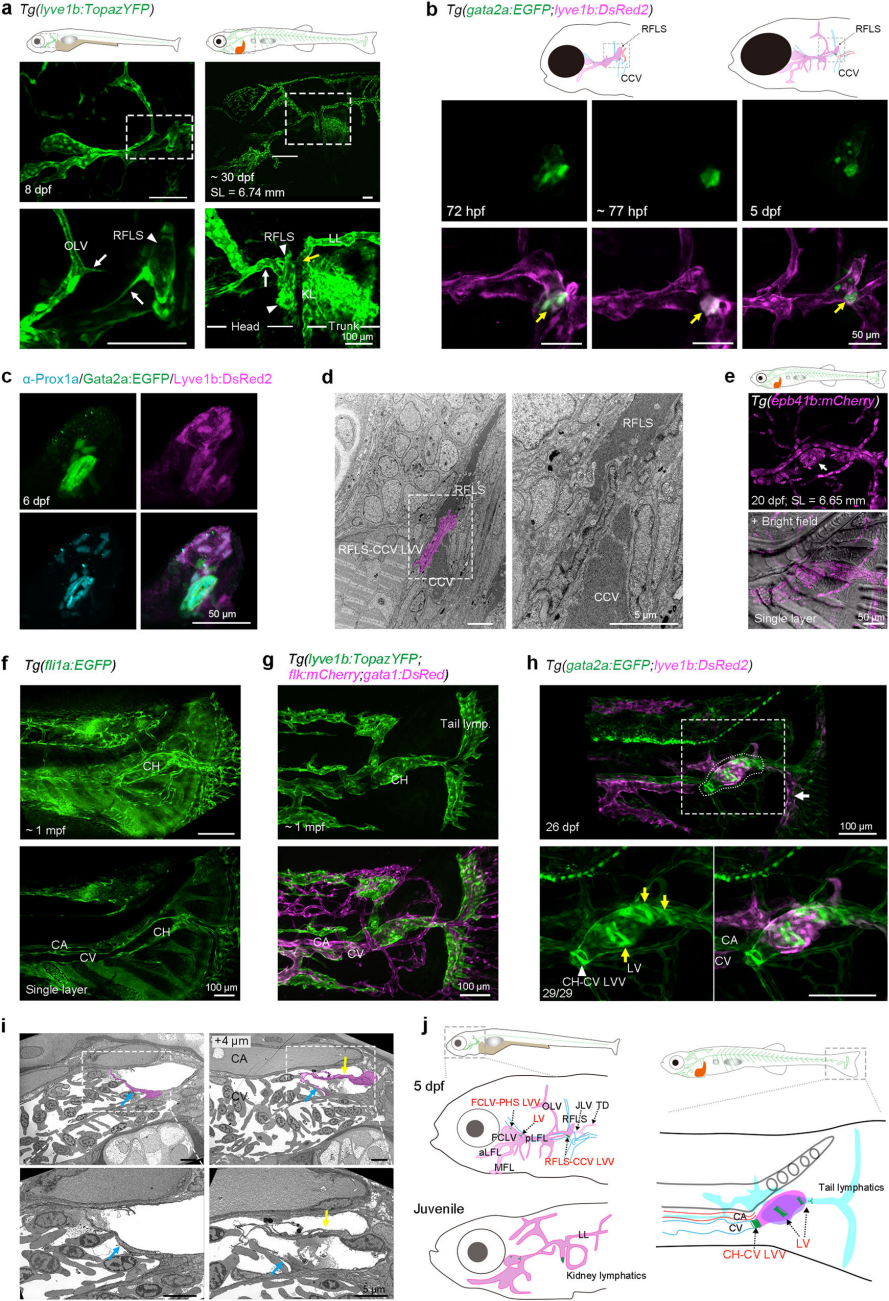
New valves in the zebrafish lymphatic system. **a** Structure of the remaining facial lymphatic sprout (RFLS, arrowheads) during zebrafish larvae development as labeled by *Tg(lyve1b:TopazYFP)*. The white and yellow arrows indicate the sprouts or connections of the RFLS with the otolithic lymphatic vessel (OLV) and trunk superficial lateral lymphatic vessels (LL), lymphatics around the kidney (KL), respectively. Bottom, enlarged images of the boxed regions in the upper panels. SL, standard length. Scale bars, 100 μm. **b** Development of the RFLS-CCV LVV (yellow arrows) labeled with *gata2a:EGFP* at different developmental stages. Uncropped images are presented in Supplementary Fig. 1b. Scale bars, 50 μm. **c** Prox1a immunostaining for the RFLS-CCV LVV in *Tg(gata2a:EGFP;lyve1b:DsRed2)* transgenic larvae at 6 dpf. Uncropped images are presented in Supplementary Fig. 1c. **d** Transmission electron microscopy shows the valve (magenta) structure at the RFLS-CCV conjunction site. The boxed region is enlarged at the right panel. CCV, common cardinal vein. Scale bars, 5 μm. **e** The existence of red blood cells (arrow) with *epb41b:mCherry* expression in the caudal heart (CH). Scale bar, 50 μm. **f** The CH structure is labeled by *fli1a:EGFP*, and a single-layer picture in the bottom panel shows the connection between the CH and CV. CH, caudal heart; CV, caudal vein; CA, caudal artery. Scale bars, 100 μm. **g** The CH structure is labeled by *lyve1b:TopazYFP* but not *flk:mCherry*. *gata1:DsRed* is used to identify certain red blood cells in the CH. Note that the tail lymphatics are blind-ended. **h** the CH-LVs and CH-CV LVV labeled by *gata2a:EGFP* in the CH structure at 26 dpf. The CH (the region with dotted lines) is labeled by *lyve1b:DsRed2*. Bottom, enlarged images of the boxed region in the upper panel. The white arrow indicates tail lymphatic vessels, yellow arrows indicate lymphatic valves in the caudal heart (CH-LVs), and the white arrowhead indicates the lymphovenous valve that connects the caudal heart and caudal vein (CH-CV LVV). Scale bars, 100 μm. **i** Transmission electron microscopy shows the lymphovenous valve (magenta) at the CH-CV connection site. Two ultra-thin sections 4 μm apart were chosen to show the two leaflets (the blue and yellow arrows) of this valve structure. The box regions are enlarged at the bottom panel. Scale bars, 5 μm. **j** Left panel, schematic diagram of valves in the facial lymphatic vasculature (purple) at 5 dpf and juvenile stage. The JLV connects with both thoracic (TD) and kidney lymphatics. Blue, vein; red, artery; green, valves. Right panel, schematic diagram of valves in the tail lymphatic vasculature. Purple, caudal heart; blue, caudal vein (CV); red, caudal artery (CA); green, valves; cyan, lymphatic vasculature. All the images are anterior to the left, dorsal upward

The facial lymphatic sprouts (FLS) that arise from the common cardinal vein (CCV) contribute to the posterior region of FLVs at 2 dpf, and then lose the connection to the lateral facial lymphatic vessels (LFLs) at 5 dpf.^53–55^ However, continuous observation in *Tg(lyve1b:TopazYFP)* transgenic fish, which labels lymphatic and venous vessels, revealed that the FLS did not disappear after 5 dpf, but continued to grow and connected with both the otolithic lymphatic vessel (OLV), the trunk superficial lateral lymphatic vessels (LLs), and lymphatics around kidney (KLs) (Fig. 1a, Supplementary Fig. 1a and Supplementary Movie 1, 2). As the FLS exhibits morphological changes during lymphatic development, we named this structure the remaining FLS (RFLS) after 3 dpf. Given that the FLS/RFLS contributes to FLVs and connects with the CCV, we assumed that there may exist an LVV between the RFLS/FLS and the CCV. By carefully observing *Tg(gata2a:EGFP;lyve1b:DsRed2)* transgenic fish,^56^ we noted that some cells located at the junction between the RFLS and CCV expressed *gata2a:EGFP* fluorescent protein from 72 hpf onward and became much more condensed by 77 hpf and 5 dpf (Fig. 1b and Supplementary Fig. 1b). By detecting Prox1a expression, we found that, compared to the moderate level of Prox1a in the LECs, these cells displayed relatively high levels of Prox1a (Prox1a^hi^) expression at 6 dpf (Fig. 1c and Supplementary Fig. 1c), which is a characteristic of valve cells.^9,10^ Using transmission electron microscopy (TEM), we further confirmed the existence of a structure that prevented direct connection of RFLS to CCV (Fig. 1d). When observing the dynamic behaviors of this presumed valve structure, we found that the leaflet motion of RFLS-CCV LVVs was similar to that of FCLV-PHS LVVs discovered previously at the junction between facial collecting lymphatic vessels (FCLVs) and the primary head sinus (PHS)^13^ (Supplementary Movie 3). Therefore, we conclude that, in addition to the FCLV-PHS LVVs, another pair of LVVs is present at the junction of the RFLS and CCV, referred to as RFLS-CCV LVVs in the head region (Fig. 1j).

Consistent with the findings in the resting glass catfish (*Kryptopterus bicirrhis*),^57^ we found that blood from the caudal fin indeed flowed through the caudal heart (CH), an interesting structure located under the tail notochord,^57,58^ into the caudal vein (CV) in the zebrafish (Supplementary Movie 4). And we could clearly observe that red blood cells labeled with *epb41b:mCherry* flowed through the tail lymphatic vessels into the CV by passing the CH in some *Tg(epb41b:mCherry)* fish and this would be much more obvious after stress or handling of fish (Fig. 1e and Supplementary Movie 4). However, to our surprise, as observed in different transgenic backgrounds, the caudal heart structure was *fli1a:EGFP*, labeling endothelial cells, and *lyve1b:TopazYFP* positive, but *flk:mCherry*, labeling blood endothelial cells only, negative. Thus, that structure could be described as a lymphatic-like structure, which connects the tail lymphatic vessels with both the caudal vein (CV) and the trunk lymphatic vessels, and might receive blood from tail structure under stress conditions (Fig. 1f, g and Supplementary Movie 5, 6). By injecting 2000 kDa Dextran-Rhodamine into the tail lymphatic vessels to imitate the lymph, we further observed direct link between the CH and CV (Supplementary Movie 7). We performed retro-orbital injection of 2000 kDa Dextran-Fluorescein to label the circulation and found the existence of caudal heart in adult fish (Supplementary Fig. 1d). The caudal heart of adult fish also uniquely expressed lymphatic marker *lyve1b:TopazYFP* (or *lyve1b:DsRed2*), but not blood vascular marker *flk:mCherry* (or *flk:EGFP*), indicating CH is a lymphatic-like structure (Supplementary Fig. 1e). Moreover, a number of bicuspid valve structures labeled with *gata2a:EGFP* were discovered in the CH region, including several LVs in CH and a specific LVV at the junction between the CH and CV, referred to as the CH-LVs and CH-CV LVV, respectively (Fig. 1h and Supplementary Movie 8, 9). We also confirmed the valve structure of CH-CV LVV by transmission election microscopy (Fig. 1i and Supplementary Fig. 1f). Taken together, these data provide the first evidence of valve structures in the trunk region of zebrafish (Fig. 1j).

### Specific repression of Erk activity in FCLV during valve cell specification

**Fig. 2:**
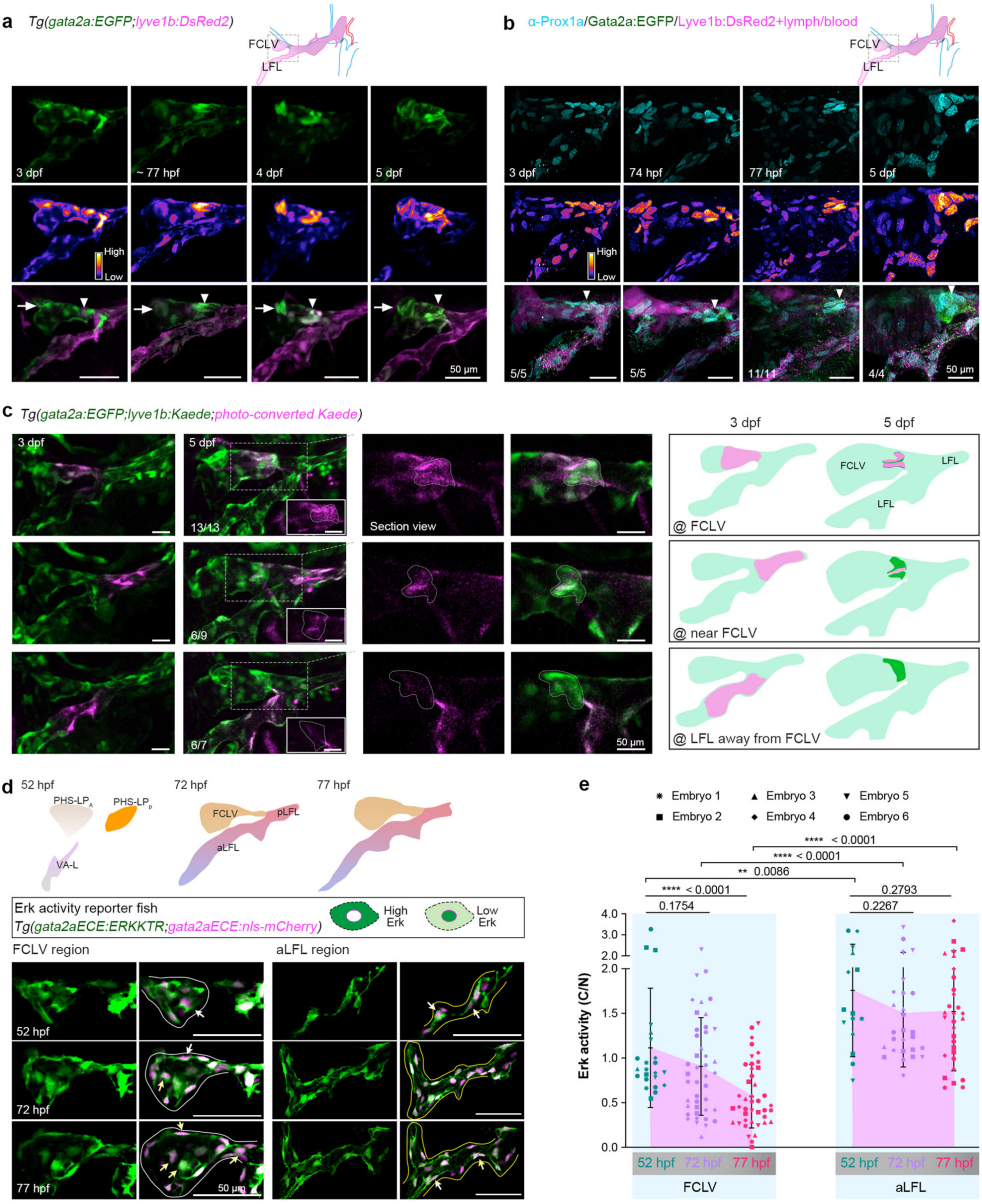
Repressed Erk activity in FCLVs during valve formation. **a** Development of the lymphatic valve (arrowheads) and FCLV-PHS LVV (arrows) labeled with *gata2a:EGFP* in the anterior part of facial lymphatic vessels (dashed box) at different developmental stages. Middle panel shows *gata2a:EGFP* expression presented in Fire LUT (Fiji). The arrowheads indicate the putative valve-forming sites. Scale bars, 50 μm. **b** Detection of valve-forming LECs labeled by high expression of Prox1a at different developmental stages. Middle panel shows Prox1a expression presented in Fire LUT (Fiji). The numbers of embryos with the Prox1a expression pattern (cyan) are indicated. Arrowheads point out valve-forming regions. Scale bars, 50 μm. **c** Lineage tracing of the valve cells from the surrounding lymphatic vessels with photoconverted Kaede. The LECs at FCLV, LFL near the FCLV, or LFL away from the FCLV were photoconverted at 3 dpf and the valve formation was observed at 5 dpf. The middle two panels are the enlarged and section views of the boxed regions on the left, and valve structures are labeled by dotted lines. The numbers of embryos with the indicated phenotype are shown. Photoconversion results are summarized as schematic drawings on the right. Scale bars, 50 μm. **d** Upper panel depicts the formation of anterior facial lymphatic vessels. Two populations of LEC precursors from PHS, PHS-LP_A_ and PHS-LP_P_, contribute to the FCLV, and LEC precursors from ventral aorta, VA-L, contribute to the anterior part of LFL (aLFL) along with PHS-LP_P_. Lower panels, Erk activity detected by ERKKTR biosensor in *Tg(gata2aECE:ERKKTR;gata2aECE:nls-mCherry)* fish during lymphatic development, from 52 hpf to 77 hpf. The FCLV region (white solid lines) and anterior part of the LFL (aLFL, yellow solid line) are shown. White arrows indicate cells with high Erk activity, and yellow arrows indicate cells with relative low Erk activity. The individual cells used for ERKKTR-EGFP analysis are listed in Supplementary Fig. 2c, d. Scale bars, 50 μm. **e** Quantification of the cytoplasmic/nuclear (C/N) intensity ratios used as readout for Erk activity in individual cells. Unpaired two-tailed *t* test, *n* = 2 independent experiments, one of them is shown here. All images are anterior to the left, dorsal upward

In *Tg(gata2a:EGFP;lyve1b:DsRed2)* fish, we observed a specific accumulation of cells with high-level expression of *gata2a:EGFP* in the FCLV but not the LFL, and then at the valve-forming region around 3 dpf (LVV at 72 hpf and LV at 77 hpf), which develops into the bicuspid valve structure over the next 2 days (Fig. 2a). Prox1a^hi^ expression at different stages validated lymphatic valve development. The FCLV accumulated LECs with Prox1a^hi^ expression about 3 dpf ( ~ 77 hpf) at valve sites, indicating valve-forming LEC specification (Fig. 2b).

Next, *Tg(lyve1b:Kaede)* transgenic fish were used to track valve-forming LEC emergence and lineage specification in LVs using photoconvertible labeling and in vivo time-lapse photography. Photoconverting LECs in the FCLV and LFL at 3 dpf showed that most FCLV-LV cells at 5 dpf came from the FCLV (Fig. 2c). Although a small proportion of LFL LECs near the FCLV contributed to the valve structure, LFL LECs slightly distant from the FCLV did not differentiate into valve-forming LECs (Fig. 2c), which is consistent with previous reports that valve-forming LECs derive from lymphatic vessels in anatomical proximity to the LV structure.^10,59,60^ Thus, valve-forming LECs are mostly FCLV-derived at 3 dpf.

Vegfc-Vegfr3-mediated activation of Erk signaling is vital to lymphatic endothelial cell (LEC) specification and migration.^23,24^ However, it has not been described how Erk signaling is dynamically controlled throughout lymphatic development, particularly in valve specification. As previously reported, the ERK-KTR (ERK kinase translocation reporter) biosensor could be used to determine Erk signaling activity by measuring the cytoplasmic/nuclear (C/N) signal ratio.^61^ Though ERK-KTR system has been used in zebrafish,^62–64^ we also generated a transgenic line *Tg(ef1a:ERKKTR)* that expresses the ERK-KTR biosensor ubiquitously to evaluate its responsiveness in our hands. Erk activity was found to be higher in most cells in the embryonic margin (Supplementary Fig. 2a), which is similar with the distribution of p-Erk reported previously.^65^ When embryos were incubated in the MEK inhibitor selumetinib from sphere stage onwards, this unique pattern of Erk activity disappeared (Supplementary Fig. 2a), shortening the anterior-posterior axis^66^ and disrupting the somite boundary formation^63^ (Supplementary Fig. 2b). Therefore, the Erk activity can be monitored by the ERK-KTR biosensor.

We created another ERK-KTR biosensor transgenic line, *Tg(gata2aECE:ERKKTR)*, driven by *gata2a* endothelial core element (ECE),^13^ to measure LEC Erk activity at the single cell level with high temporal resolution. By visualizing the ERK-KTR biosensor with nuclear marker *Tg(gata2aECE:nls-mCherry)*, we spatiotemporally evaluated LEC ERK-KTR C/N ratios. Using two distinct statistical approaches, we consistently observed a slight decrease in Erk activity in both FCLV and aLFL LECs from 52 to 72 hpf. However, a distinct reduction in Erk activity was only evident in FCLV LECs from 72 to 77 hpf during valve development, whereas it remained relatively unchanged in aLFL LECs (Fig. 2d, e, Supplementary Fig. 2c–f, and Supplementary Movie 10). We further used selumetinib to inhibit MEK activity from 56 hpf to test the responsiveness of this reporter line in lymphatic vessels. After inhibition, aLFL Erk activity and p-Erk protein level were found to be significantly reduced (Supplementary Fig. 2g–i). Furthermore, we applied time-lapse imaging on Erk reporter fish from 72 to 78 hpf. The results showed a more consistent increase of nuclear fluorescence intensity and corresponding decrease of ERKKTR intensity ratio of cytoplasmic to nuclear (C/N) fluorescence in FCLV LECs than in aLFL LECs (Supplementary Fig. 3a–c, and Supplementary Movie 11), indicating a decrease of Erk activity in FCLV LECs. This observation was supported by p-Erk immunostaining, which revealed that the p-Erk signal was clearly suppressed in the FCLV region compared to the aLFL region and decreased further in FCLV LECs at 77 hpf (Supplementary Fig. 4). We hypothesize that FCLV Erk suppression is necessary for the initiation of *gata2a*-marked valve-forming LEC program and subsequently valve formation.

### Depleting *rasa1* impairs valve-forming cell specification

Rasa1 is a Ras GTPase-activating protein (RasGAP) that accelerates conversion of Ras protein from an active GTP-bound state back to an inactive GDP-bound state, hence acting as a negative regulator of Erk signaling.^46^ It has been shown that loss-of-function *Rasa1* mutations in mouse embryos likewise result in impaired valve development, and administration of ERK inhibitor AZD6244 partially rescues LVV development in the absence of RASA1 in mice.^45,47^ However, it is yet uncertain if Erk signaling has to be suppressed by Rasa1 during the process of valve formation.

**Fig. 3:**
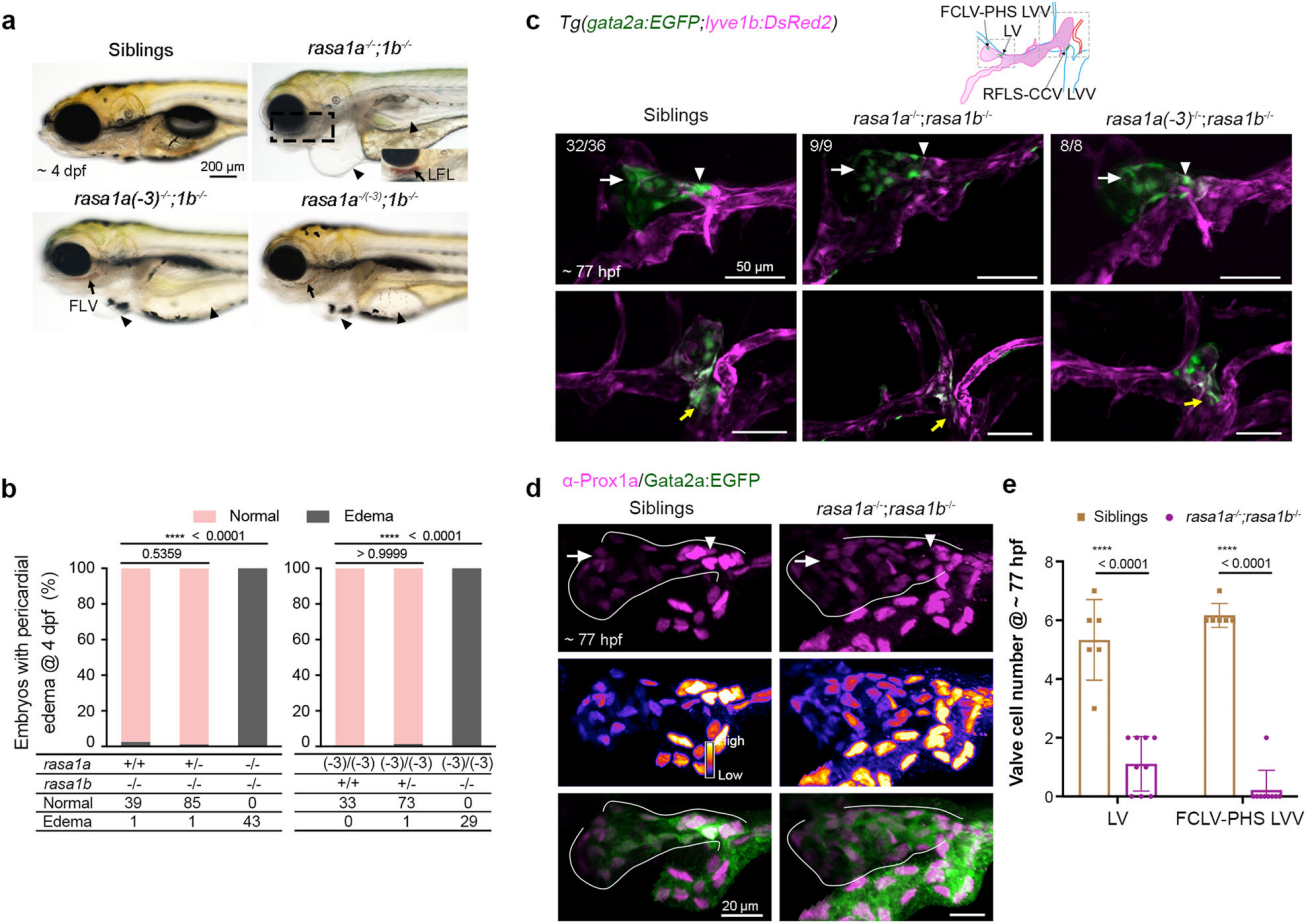
*rasa1* mutants have defects in lymphatic valve specification. **a** Double mutants of *rasa1a* and *rasa1b* exhibit pericardial edema (arrowheads) and blood filling of facial lymphatic vessels (arrows) at 4 dpf. A lateral-ventral view of the LFL in the boxed region is shown. *Casper* background was introduced into *rasa1a*^−/−^*;rasa1b*^−/−^ mutants to facilitate observation. Other mutants were on Tubingen background. Scale bar, 200 μm. **b** Statistical summary of pericardial edema in the offspring of *rasa1a*^−/+^*;rasa1b*^−/−^ and *rasa1a(-3)*^−/−^*;rasa1b*^−/+^ mutants intercrossing. Two-sided Fisher’s exact test. **c** Lack of lymphatic valve (arrowheads), FCLV-PHS LVV (arrows), and RFLS-CCV LVV (yellow arrows) at 77 hpf in *rasa1a*^−/−^*;rasa1b*^−/−^ and *rasa1a(-3)*^−/−^*;rasa1b*^−/−^ double mutants. The numbers of embryos with the indicated valve morphology are shown. Scale bars, 50 μm. **d** Prox1a immunostaining (magenta) in siblings and *rasa1a*^−/−^*;rasa1b*^−/−^ double mutants on the *Tg(gata2a:EGFP*) background at 77 hpf. Middle panel shows Prox1a expression presented in Fire LUT (Fiji). Scale bars, 20 μm. **e** Statistical analyses of the valve-forming LECs in siblings (*n* = 6) and *rasa1a*^−/−^*;rasa1b*^−/−^ mutants (*n* = 9) in (**d**). Unpaired two-tailed *t* test

Genomic duplication in teleost fish^67^ resulted in two *rasa1* genes, *rasa1a* and *rasa1b*, in the zebrafish. We first generated three types of *rasa1* mutants separately with CRISPR/Cas9 technology, *rasa1a*^*tsu38*^ (*rasa1a*^−/−^) with a 2-bp deletion and 18-bp insertion, *rasa1a*^*tsu40*^ (*rasa1a(-3)*^*−/−*^) with a 3-bp deletion, and *rasa1b*^*tsu39*^ (*rasa1b*^−/−^) with a 308-bp deletion and 31-bp insertion (Supplementary Fig. 5a), and found that all of them developed normally to adulthood without noticeable morphological abnormalities, possibly due to compensatory function of Rasa1a and Rasa1b. We next created *rasa1a*^−/−^;*rasa1b*^−/−^, *rasa1a(-3)*^−/−^;*rasa1b*^−/−^ and *rasa1a*^−/*(−3)*^;*rasa1b*^−/−^ double mutants to examine phenotypic changes. All of them exhibited blood filling within lymphatic vessels with significant pericardial edema at 4 dpf (Fig. 3a, b). It is worth mentioning that, the 3-bp deletion in *rasa1a(-3)*^*−/−*^ mutant allele is essential for its functions in lymphatic valve formation. We found an overgrowth of lymphatic vessels at 77 hpf in *rasa1a*^−/−^;*rasa1b*^−/−^ double mutants in *Tg(flk:mCherry;lyve1b:TopazYFP)* transgenic background, but no obvious abnormities in blood vessel formation (Supplementary Fig. 5b). However, LV and LVV valve cells with *gata2a:EGFP* expression at vessel bifurcation were nearly absent in *Tg(gata2a:EGFP;lyve1b:DsRed2)* transgenic double mutants, although *gata2a:EGFP* positive valve-forming LECs were still detectable in FCLVs (Fig. 3c). The Prox1a immunostaining showed fewer and disorganized Prox1a^hi^ valve cell clusters in *rasa1a*^*−/−*^*;rasa1b*^*−/−*^ mutants (Fig. 3d, e). These results indicate that *rasa1* is necessary for the specification of valve-forming cells at vessel bifurcation sites.

**Fig. 4:**
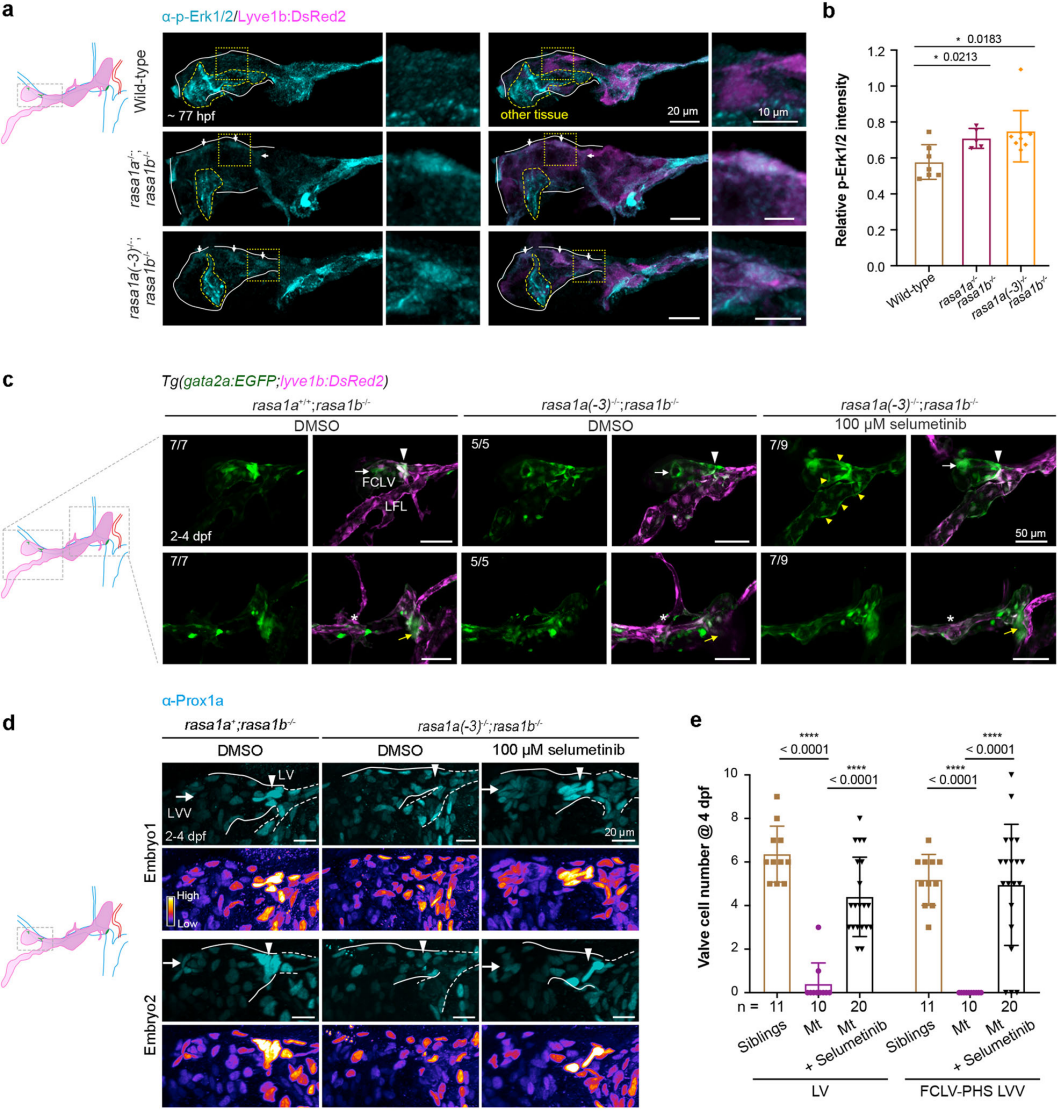
*rasa1* regulates lymphatic valve specification through inhibiting Erk signaling. **a** p-Erk1/2 immunostaining reveals increased Erk signal in FCLV LECs in *rasa1a*^−/−^*;rasa1b*^−/−^ and *rasa1a(-3)*^−/−^;*rasa1b*^−/−^ mutant embryos at 77 hpf. Solid lines, FCLV; yellow dashed lines, other tissues with p-Erk1/2 signals. Arrows indicate the obvious p-Erk1/2 signals in FCLV. Right, enlargement of the boxed regions in the left panels. Scale bars, 20 μm (left) or 10 μm (right). **b** Relative p-Erk1/2 intensity compared to *lyve1b:DsRed2* expression in FCLV in wild-type and mutant embryos. Unpaired two-tailed *t* test (at least 3 independent experiments; Wild-type n = 7; *rasa1a*^−/−^;*rasa1b*^−/−^ *n* = 5; *rasa1a(-3)*^−/−^;*rasa1b*^−/−^ *n* = 8). **c** Selumetinib treatment from 2–4 dpf restored the valve structure in *rasa1a(-3)*^−/−^;*rasa1b*^−/−^ labeled with *gata2a:EGFP* and also induced ectopic *gata2a:EGFP* expression in the FLV. Arrowheads, LVs; arrows, FCLV-PHS LVVs; yellow arrows, RFLS-CCV LVVs; yellow arrowheads, ectopic *gata2a:EGFP* positive cells; asterisks, OLVs. The numbers of embryos with exhibited valve structures are shown. Scale bars, 50 μm. **d** Selumetinib treatment restores the LV and LVV formation in *rasa1a(-3)*^−/−^*;rasa1b*^−/−^ mutants at 4 dpf. Prox1a immunostaining was used to label the valve-forming LECs. Prox1a expressions were also presented in Fire LUT (Fiji). Arrowheads, LVs; arrows, FCLV-PHS LVVs. *N* = 3 independent experiments. Scale bars, 20 μm. **e** Statistical analysis of the valve-forming LECs in siblings with DMSO (*n* = 11), *rasa1a(-3)*^−/−^*;rasa1b*^−/−^ mutant (Mt) with DMSO (*n* = 10), and Mt with 100 μM selumetinib (*n* = 20) in (**d**). Unpaired two-tailed *t* test. All images are anterior to the left, dorsal upward

Using immunofluorescence, we examined p-Erk levels in *rasa1a*^−/−^*;rasa1b*^−/−^ and *rasa1a(-3)*^−/−^*;rasa1b*^−/−^ mutant larvae at 77 hpf and saw relatively higher levels of p-Erk in a subset of mutant larvae’s FCLV LECs, especially at the putative valve-forming sites, compared to wild-type ones (Fig. 4a, b). Incubation of *rasa1a(-3)*^−/−^*;rasa1b*^−/−^ mutant embryos in 100 μM selumetinib at any time from 2 to 3 dpf rescued *gata2a:EGFP*-labeled valve structures at 4 dpf (Fig. 4c and Supplementary Fig. 6), and this effect was validated by Prox1a immunostaining (Fig. 4d, e). Strikingly, compared to siblings or *rasa1a(-3)*^−/−^*;rasa1b*^−/−^ mutant embryos, selumetinib-treated mutant embryos displayed ectopically elevated *gata2a:EGFP* expression in the aLFL region with diminished *lyve1b:DsRed2* expression (Fig. 4c and Supplementary Fig. 6). Taken together, we suppose that the quiescence of Erk signaling in the FCLV promotes the initiation of valve-forming cell program with stimulation of *gata2a* expression.

### Inhibition of Erk signaling leads to valve hyperplasia

**Fig. 5:**
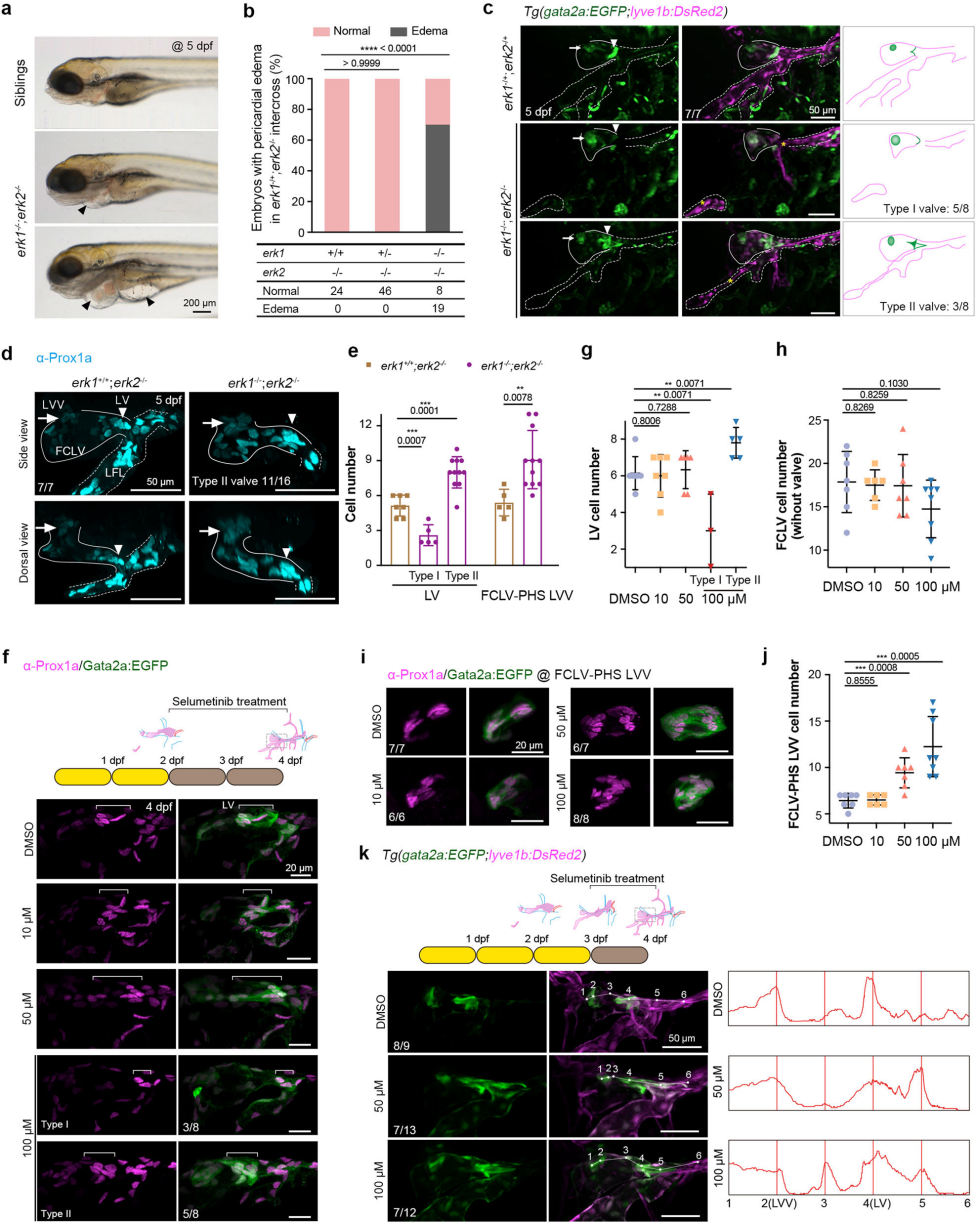
Inhibition of MAPK/Erk signaling leads to valve hyperplasia. **a** *erk1*^*−/−*^*;erk2*^*−/−*^ double mutants develop obvious edema at 5 dpf. Arrowheads indicate the edema in the heart and gut regions. Scale bar, 200 μm. **b** Statistical data for pericardial edema in the offspring of *erk1*^-/+^;*erk2*^−/−^ mutants intercrossing at 5 dpf. Two-sided Fisher’s exact test. **c** *erk1*^*−/−*^*;erk2*^*−/−*^ double mutants show defects in both lymphatic vessel and valve formation. In *erk1*^*−/−*^*;erk2*^*−/−*^ double mutants, two types of valve phenotypes are observed, which are summarized in the schematic drawings on the right. Asterisks, anterior LFLs; arrowheads, lymphatic valves; arrows, FCLV-PHS LVVs. Scale bars, 50 μm. **d** At 5 dpf, Prox1a immunostaining reveals an increase of valve cells in the FCLV-PHS LVV and type II FCLV-LV in *erk1*^*−/−*^*;erk2*^*−/−*^ double mutants. The number of mutants with type II valve is indicated. Solid lines indicate FCLV and dashed lines indicate LFL. Scale bars, 50 μm. **e** Statistical analysis of the valve-forming LECs with high Prox1a expression in *erk1*^*+/+*^*;erk2*^*−/−*^ (*n* = 7) siblings or *erk1*^*−/−*^*;erk2*^*−/−*^ (*n* = 16) mutants at 5 dpf in (**d**). Unpaired two-tailed *t* test (LV for *erk1*^*+/+*^*;erk2*^*−/−*^ *n* = 7; LV for *erk1*^*−/−*^*;erk2*^*−/−*^ *n* = 16; FCLV-LVV for *erk1*^*+/+*^*;erk2*^*−/−*^ *n* = 5; FCLV-LVV for *erk1*^*−/−*^*;erk2*^*−/−*^ n = 11). **f** Detection of the valve-forming LECs by Prox1a and *gata2a:EGFP* immunostaining in embryos treated with different doses of selumetinib from 2 to 4 dpf. Brackets indicate the LV structures. Two different types of LVs are observed. The numbers of embryos with the Prox1a expression pattern (magenta) in LVs are indicated. Scale bars, 20 μm. **g** Statistical analysis of LV cells after selumetinib treatment in (**f**). Unpaired two-tailed *t* test, (DMSO *n* = 7; 10 *n* = 7; 50 *n* = 6; 100 μM *n* = 3 and 5). **h** Statistical analysis of FCLV cells, excluding the valve-forming LECs with high Prox1a expression in (**f**). Unpaired two-tailed *t* test, (DMSO *n* = 7; 10 *n* = 6; 50 *n* = 7; 100 μM *n* = 8). **i** Detection of FCLV-PHS LVV formation in embryos treated with different doses of selumetinib from 2 to 4 dpf. The numbers of embryos with the Prox1a expression pattern (magenta) are indicated. Scale bars, 20 μm. **j** Statistical analysis of FCLV-PHS LVV cells in (**i**). Unpaired two-tailed *t* test (DMSO *n* = 7; 10 *n* = 6; 50 *n* = 7; 100 μM *n* = 8). **k** Live imaging of the ectopic valve-forming LECs labeled with *gata2a:EGFP* in embryos treated with selumetinib from 3 to 4 dpf. *gata2a:EGFP* fluorescence intensities at positions 1–6 are shown. Ectopic *gata2a:EGFP* expression was found at position 5 in 50 μM treated embryos and positions 5 and 3 in 100 μM treated embryos. All images are anterior to the left, dorsal upward

To explore Erk signaling in valve-forming cell development in further detail, we generated the *erk1*^*tsu45*^ mutant line (*erk1*^−/−^) with a 47-bp deletion and the *erk2*^*tsu46*^ mutant line (*erk2*^−/−^) with a 5-bp deletion (Supplementary Fig. 7a). Because compensatory role of Erk1 and Erk2 helps *erk1*^−/−^ and *erk2*^−/−^ single mutants reach adulthood, we created *erk1*^−/−^*;erk2*^*−/−*^ double mutants. At 5 and 6 dpf, 70% (*n* = 19/27) of these mutants had pericardial edema (Fig. 5a, b and Supplementary Fig. 7b). Compared to the morphology of *lyve1b:DsRed2*-labeled FLVs in *erk1*^+/−^*;erk2*^*+/−*^ double heterozygote larvae at 5 dpf, the aLFL, the OLV, and the thoracic duct (TD) in *erk1*^−/−^*;erk2*^*−/−*^ double mutants were defective or completely lost (Fig. 5c and Supplementary Fig. 7c, d), which were consistent with the previous report that Erk inhibition blocks trunk lymphatic sprouting and differentiation.^23^ Since Erk inhibition probably affects blood vessel formation,^68^ which would impact lymphatic development and thus valve development, we examined the vascular system of the *erk1*^−/−^*;erk2*^*−/−*^ double mutants in *Tg(lyve1b:DsRed2;gata2a:EGFP)* background at 5 dpf. Results showed that the blood vessels in *erk1*^−/−^*;erk2*^*−/−*^ double mutants could develop appropriately in the trunk regions, such as the DA, PCV (Supplementary Fig. 7d). Angiography by injecting 2000 kDa Dextran-Rhodamine into CCV also revealed normal blood circulation in *erk1*^−/−^*;erk2*^*−/−*^ double mutants (Supplementary Fig. 7e), which may be attributed to the maternal expression of *erk1* and *erk2* (Supplementary Fig. 7f). As a result, we believe that the lymphatic abnormalities observed in *erk1*^−/−^*;erk2*^*−/−*^ double mutants may be unrelated to the vascular development.

Nevertheless, the phenotypes of valve structures in *erk1*^−/−^*;erk2*^*−/−*^ double mutants are more complicated. Although *gata2a:EGFP*-positive valve-forming LECs in FCLV-PHS LVV and RFLS-PHS LVV were obviously increased compared to *erk1*^+/-^*;erk2*^*+/-*^ double heterozygote larvae, we found two distinct phenotypes of FCLV-LVs (Fig. 5c and Supplementary Fig. 7c). Briefly, type I was characterized by decreased valve-forming cell number (valve hypoplasia; *n* = 5/8) and type II by increased valve-forming cell number (valve hyperplasia; n = 3/8) (Fig. 5c). Prox1a expression was used to verify this observation. Compared to siblings, in the *erk1*^−/−^*;erk2*^*−/−*^ double mutants, the number of valve-forming LECs with Prox1a^hi^ expression increased from 5.4 to 9.1 in FCLV-PHS LVVs, fell from 5.1 to 2.6 in type I FCLV-LVs, and increased to 8.0 in type II FCLV-LVs (Fig. 5d, e). We also found that applying 100 μM selumetinib to *Tg(gata2a:EGFP)* transgenic larvae from 2 to 4 dpf well mimicked the phenotype of the *erk1*^−/−^*;erk2*^*−/−*^ double mutants, showing slightly decreased LECs with moderate Prox1a expression in FCLVs, increased numbers of valve cells with Prox1a^hi^ expression in FCLV-PHS LVVs, and two opposite changes in FCLV-LVs (Fig. 5f–j).

Since valve-forming cells in the FCLV-LV are largely derived from the FCLV LECs, it is clear that FCLV’s developmental status is critical for valve-forming cell formation in FCLV-LV. So, at 3 dpf when FCLV had formed but valve-forming LEC specification was about to begin, we applied selumetinib and found enhanced ectopic *gata2a:EGFP* expression in the FCLV surrounding FCLV-LV (Fig. 5k). This observation is consistent with ectopic *gata2a:EGFP* expression in *erk1*^*−/−*^*;erk2*^*−/−*^ mutant embryos (Supplementary Fig. 7g). Inhibition of Erk activity by selumetinib could not induce high or ectopic *gata2a:EGFP* expression in trunk blood or lymphatic vessels (Supplementary Fig. 8a, b). Thus, in *erk1*^−/−^*;erk2*^*−/−*^ double mutants or embryos treated with selumetinib from 2 dpf onwards, the bimodal impact on FCLV-LV development was likely caused by dynamic alterations in Erk signaling during lymphatic development, resulting in some embryos with deficient FLVs having valve hypoplasia and others with normal FLV formation having valve hyperplasia.

### Efnb2-Ephb4 signaling is essential for lymphatic valve formation

Since mutations in the human EFNB2-EPHB4 cassette are linked to both vascular and lymphatic diseases, and Rasa1 probably interacts with Ephb4 to inactivate Ras in endothelial cells to regulate vascular development,^38,69^ we wondered if Ephb4 and its ligand Efnb2 could inhibit Erk signaling to promote lymphatic valve specification upstream of Rasa1.

**Fig. 6:**
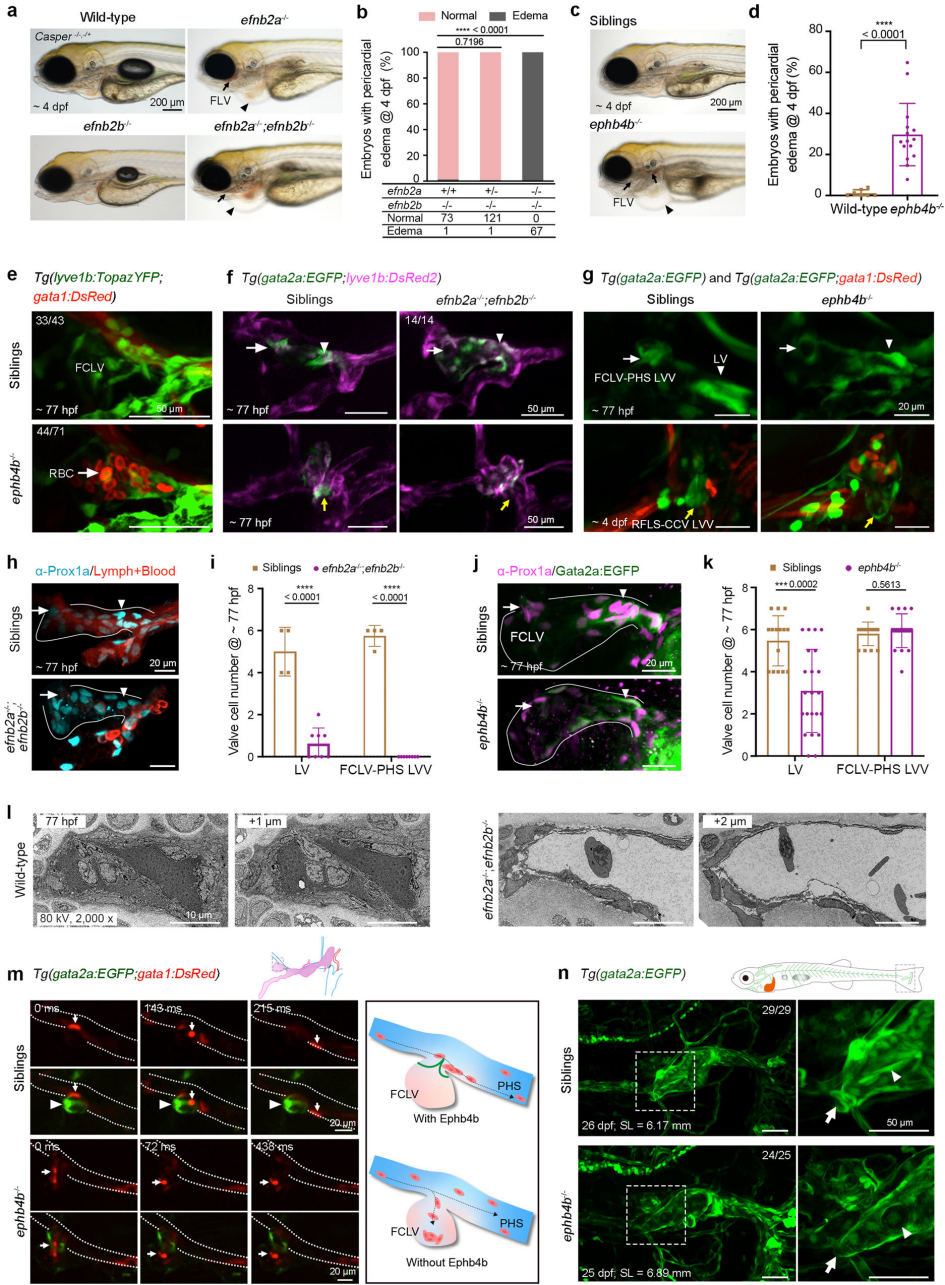
*efnb2*-*ephb4b* mutants have defects in lymphatic system. **a** *efnb2a*^−/−^ and *efnb2a*^−/−^*;efnb2b*^−/−^ double mutants exhibit pericardial edema (arrowheads) and blood filling of facial lymphatic vessels (arrows) at 4 dpf, whereas *efnb2b*^−/−^ develops normally. *Casper* background was introduced to facilitate observation. Scale bar, 200 μm. **b** Statistical summary of pericardial edema in the offspring of *efnb2a*^-/+^*;efnb2b*^−/−^ mutants intercrossing. Two-sided Fisher’s exact test. **c** Pericardial and gut edema (arrowhead) with blood filling of lymphatic vessels (arrows) phenotype in *ephb4b*^−/−^ mutants. *Casper* background was introduced to facilitate observation. Scale bar, 200 μm. **d** Percentage of *ephb4b*^−/−^ mutants with pericardial edema. Each dot represents the percentage of edema embryos (total embryos >40) from one pair of fish with the indicated genotype. Unpaired two-tailed *t* test (WT *n* = 8; *ephb4b*^−/−^ *n* = 15). **e** *gata1:DsRed* labeled red blood cells (arrows) enter the *lyve1b:TopazYFP* labeled facial lymphatic vessel (green) at 77 hpf in *ephb4b*^−/−^ mutants. The numbers of embryos with indicated phenotype are shown. Scale bars, 50 μm. Uncropped images are presented in Supplementary Fig. 9k. **f** Lack of the lymphatic valve (arrowhead), FCLV-PHS LVV (arrow), and RFLS-CCV LVV (yellow arrow) at 77 hpf in *efnb2a*^−/−^*;efnb2b*^−/−^ double mutants. The numbers of embryos with the indicated valve morphology are shown. Scale bars, 50 μm. **g** Top, *ephb4b*^−/−^ mutants have defective LVs (arrowheads) and FCLV-PHS LVVs (arrows) at 77 hpf. Bottom, *ephb4b*^−/−^ mutants have defective RFLS-CCV LVVs (yellow arrows) and red blood cells (RBCs) entry of the RFLS at 4 dpf. Scale bars, 20 μm. **h**, Immunofluorescence of Prox1a in siblings and *efnb2a*^−/−^*;efnb2b*^−/−^ mutants at 77 hpf. The blood and lymphatic fluid have spontaneous red fluorescence. Scale bars, 20 μm. **i** Statistical analyses of the valve-forming LECs in siblings (*n* = 4) and *efnb2a*^−/−^*;efnb2b*^−/−^ mutants (*n* = 8) in (**h**). Unpaired two-tailed t test. **j** Immunofluorescence of Prox1a in siblings and *ephb4b*^−/−^ mutants on the *Tg(gata2a:EGFP)* transgenic background at 77 hpf. Arrows, FCLV-PHS LVVs; arrowheads, LVs. Valve-forming LECs are labeled by high Prox1a expression. Scale bars, 20 μm. **k** Statistical analyses of the valve-forming LECs in siblings (*n* = 15) and *ephb4b*^−/−^ mutants (*n* = 22) in (**j**). Unpaired two-tailed *t* test. **l** Transmission electron microscopy reveals the absence of LVs in *efnb2a*^−/−^*;efnb2b*^−/−^ mutants at 77 hpf. Scale bars, 10 μm. **m** Confocal imaging of RBC flow around the FCLV-PHS LVV in siblings and *ephb4b*^−/−^ mutants on the *Tg(gata2a:EGFP;gata1:DsRed)* background. Primary head sinus (PHS) is marked by white dotted lines. RBCs (arrows) are labeled by *gata1:DsRed*, and FCLV-PHS LVVs (arrowheads) are *gata2a:EGFP*-positive. RBCs enter the FCLV through the defective LVV in *ephb4b*^−/−^ mutants, but are blocked by the well-formed LVV in siblings at 77 hpf. Right, diagrams of RBC flow in the siblings and *ephb4b*^−/−^ mutants. Scale bars, 20 μm. **n** LV and LVV defects in the caudal heart in *ephb4b*^−/−^ mutants. Right, enlarged caudal hearts in the boxed regions. Arrow, CH-CV LVV; arrowhead, LV in the caudal heart. The numbers of embryos with indicated valve morphology are shown. SL, standard length. Scale bars, 50 μm. All images are anterior to the left, dorsal upward

The zebrafish genome has two *efnb2* and two *ephb4* genes. We first created unique mutant lines with matched genes (Supplementary Fig. 9a, b). Homozygous *efnb2a*^*tsu41*^ (*efnb2a*^−/−^) mutants had caudal plexus vascular malformation, blood reflux into lymphatic vessels, and edema phenotype at 4 dpf (Fig. 6a and Supplementary Fig. 9c, d), whereas *efnb2b*^*tsu42*^ (*efnb2b*^−/−^) mutants developed normally (Fig. 6a, b and Supplementary Fig. 9d). By crossing double heterozygous fish, we obtained *efnb2a*^−/−^;*efnb2b*^−/−^ double mutants. All the double mutants had lymphatic blood filling and pericardial edema defects (Fig. 6a, b), which were much more severe than *efnb2a*^−/−^ single mutants, implying that *efnb2b* may compensate for *efnb2a*. Although *efnb2a*^−/−^ and *efnb2a*^−/−^;*efnb2b*^−/−^ double mutants displayed similar blood vessel defects as reported before,^70^ their facial lymphatic vessels still formed normally (Supplementary Fig. 9e).

For *ephb4* genes, *ephb4b*^*tsu25*^ (*ephb4b*^−/−^) mutants^71^ showed similar lymphatic abnormalities at 4 dpf as did *efnb2a*^−/−^ mutants, whereas the *ephb4a*^*tsu37*^ (*ephb4a*^−/−^) mutant only displayed apparent defects in caudal plexus formation at 2 dpf, and none of them had red blood cells in the lymphatic vessels (Fig. 6c, d and Supplementary Fig. 9f). However, we found the blood circulation system was almost normal in *ephb4b*^*−/−*^ mutants (Supplementary Fig. 9g, h). These findings suggest that *ephb4a* and *ephb4b* most likely regulate blood vascular and lymphatic system development dependently. Although *ephb4b*^−/−^ mutants lived to adulthood, they had substantial hemorrhage-like defects at juvenile and adult stages, sometimes accompanied with scale protrusion and abdominal hydrops (Supplementary Fig. 9i, j). In *Tg(lyve1b:TopazYFP;gata1:DsRed2)* transgenic fish, *gata1:DsRed*-positive blood cells aggregated in the mutant lymphatic vessels from larvae to juveniles (Fig. 6e and Supplementary Fig. 9k, l), implying a persistent lymphatic system abnormality.

By generating *efnb2a*^*−/−*^;*efnb2b*^*−/−*^, *ephb4b*^*−/−*^ mutants on the *Tg(gata2a:EGFP;lyve1b:DsRed2)* or *Tg(gata2a:EGFP;gata1:DsRed)* transgenic background, we found that the LVs and LVVs in these mutants were defective (Fig. 6f, g). Immunostaining showed that *efnb2a*^*−/−*^;*efnb2b*^*−/−*^ and *ephb4b*^*−/−*^ mutants had significantly less Prox1a^hi^ cells aggregated at putative valve locations than sibling embryos, indicating a failure of valve formation (Fig. 6h–k and Supplementary Movie 12). TEM revealed a lack of LVs in *efnb2a*^*−/−*^;*efnb2b*^*−/−*^ mutants (Fig. 6l). Confocal live imaging was used to record blood flow around the FCLV-PHS LVV in the *ephb4b*^*−/−*^ mutant on the *Tg(gata2a:EGFP;gata1:DsRed)* background. In siblings at 4 dpf, *gata2a:EGFP-*expressing LVVs prevented *gata1:DsRed-*expressing red blood cells in the PHS from entering the FCLV (*n* = 7; Fig. 6m and Supplementary Movie 13). However, in *ephb4b*^*−/−*^ mutants, red blood cells in the PHS were able to flow into the FCLV through the intervening space of the defective FCLV-PHS LVV (*n* = 6; Fig. 6m and Supplementary Movie 13). In addition to the valve defects in the head region, we also observed CH-LV and CH-CV LVV defects in the trunk region of the mutants at juvenile stages (Fig. 6n). Without CH-CV LVVs, red blood cells in the CV could flow back into the CH (*n* = 3; Supplementary Fig. 10 and Supplementary Movie 14). Therefore, our findings suggest that the Efnb2-Ephb4 pathway participates in valve development.

### Inhibition of Erk signaling rescues the valve-forming cell specification in Efnb2-Ephb4 mutations

**Fig. 7:**
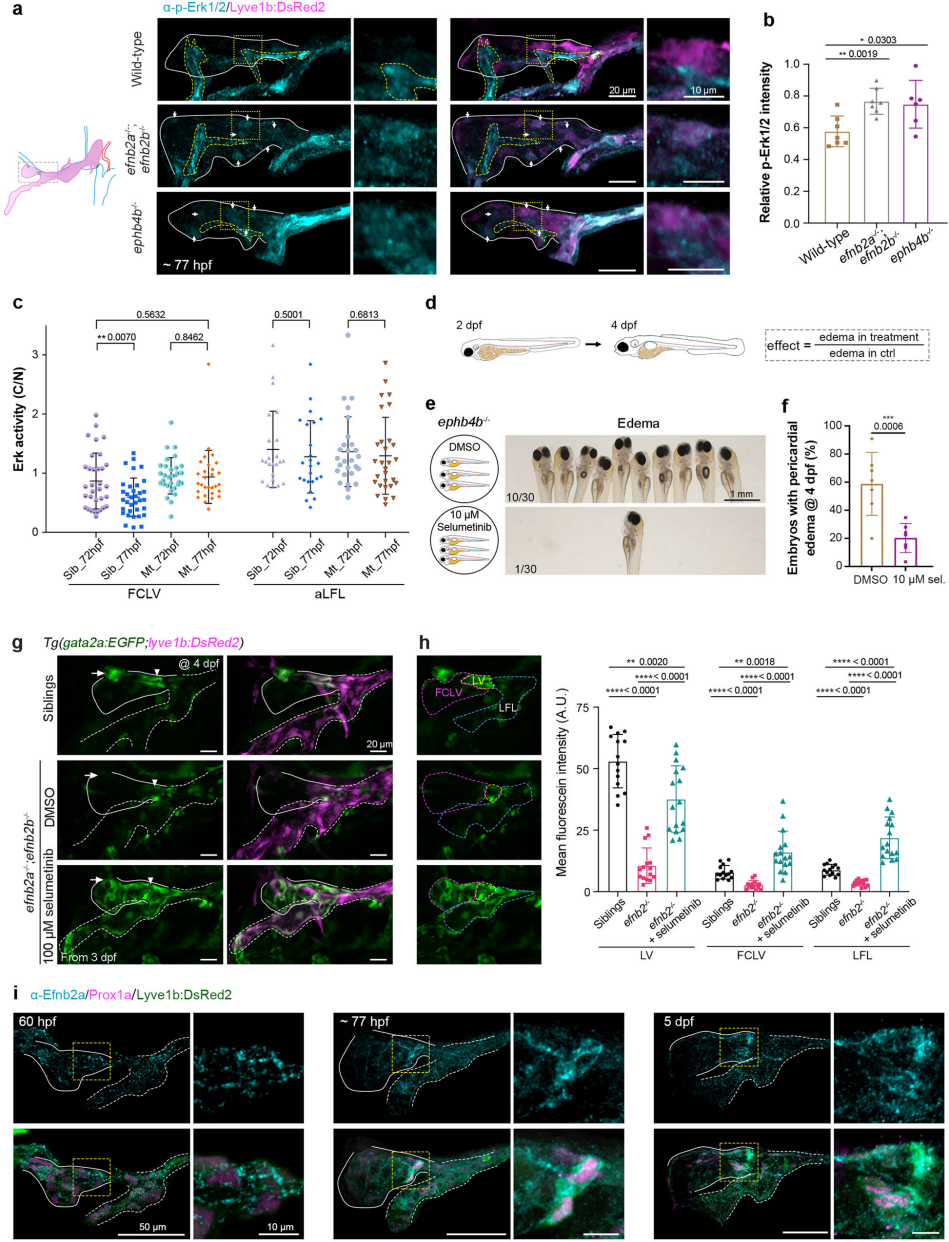
*efnb2*-*ephb4* regulate valve specification through inhibiting Erk signaling. **a** p-Erk1/2 immunostaining reveals increased Erk signal in FCLV LECs in *efnb2a*^*−/−*^*;efnb2b*^*−/−*^ and *ephb4b*^−/−^ mutant embryos at 77 hpf. Solid lines, FCLV; yellow dashed lines, other tissues with p-Erk1/2 signals. Arrows indicate the obvious p-Erk1/2 signals in FCLV. Right, enlargement of the boxed regions in the left panels. Scale bars, 20 μm (left) or 10 μm (right). **b** Relative p-Erk1/2 intensity compared to *lyve1b:DsRed2* expression in FCLV in wild-type and mutant embryos. The same batch of experiments with Fig. 4a. Unpaired two-tailed *t* test (at least 3 independent experiments; wild-type *n* = 7; *efnb2a*^*−/−*^*;efnb2b*^*−/−*^ *n* = 7; *ephb4b*^−/−^ *n* = 6;). **c** The changes of Erk activity in the FCLV and aLFL LECs of siblings (Sib) and *efnb2a*^*−/−*^*;efnb2b*^*−/−*^ mutants (Mt) during the valve-forming cell formation. Siblings, *n* = 7; *efnb2a*^*−/−*^*;efnb2b*^*−/−*^, *n* = 7 for the FCLV and *n* = 9 for the aLFL. Represented images are shown in Supplementary Fig. 11a, b. Unpaired two-tailed *t* test, *n* = 2 independent experiments, one of them is shown here. **d**, Rescue strategy using small molecules. Treatments started from 2 dpf in 6-well plates and continued until 4 dpf. **e** Representative results of the treatment of *ephb4b*^−/−^ mutant larvae with DMSO or 10 μM selumetinib. Selumetinib treatment can efficiently reduce the number of embryos with pericardial edema. Uncropped images can be found in Supplementary Fig. 11c. Scale bar, 1 mm. **f** The MEK inhibitor selumetinib can restore the pericardial edema defect in *ephb4b*^−/−^ mutants at 4 dpf. Paired two-tailed *t* test (at least 3 independent experiments; *n* = 7). **g** Selumetinib treatment from 3 dpf induces more *gata2a:EGFP* positive cells in both the FCLV and aLFL in *efnb2a*^−/−^*;efnb2b*^−/−^ mutants. Arrowheads, LVs; arrows, FCLV-PHS LVVs. Scale bars, 20 μm. **h** EGFP fluorescence intensity analyses in (**g**). FCLV, LV and LFL regions for mean intensity analyses are indicated. Unpaired two-tailed *t* test. **i** The dynamic expression of Efnb2a on the plasma membrane of FCLV LECs at 60 hpf, valve-forming cells at 77 hpf, and valve cells at 5 dpf during lymphatic valve development. Solid lines, FCLV; dashed lines, LFL. Scale bars, 50 μm (left) or 10 μm (right)

To determine whether Efnb2-Ephb4 can inhibit Erk signaling to enhance lymphatic valve specification upstream of Rasa1, we assessed p-Erk levels in *efnb2a*^−/−^;*efnb2b*^−/−^ and *ephb4b*^−/−^ mutant larvae at 77 hpf using immunofluorescence, and found that they were relatively higher in these two mutants (Fig. 7a, b), which was similar to the changes in *rasa1a(-3)*^−/−^*;rasa1b*^−/−^ mutants (Fig. 4a). Then, *Tg(gata2aECE:ERKKTR;gata2aECE:nls-mCherry)* transgenic background was introduced into *efnb2a*^*+/−*^*;efnb2b*^*+/−*^ double heterozygotes. By intercrossing *efnb2a*^*+/−*^*;efnb2b*^*+/−*^, we found that the Erk activity was not further down-regulated in the FCLV LECs of *efnb2a*^*−/−*^;*efnb2b*^*−/−*^ double mutants as it was in siblings during the valve-forming cell formation (Fig. 7 and Supplementary Fig. 11a, b).

**Fig. 8:**
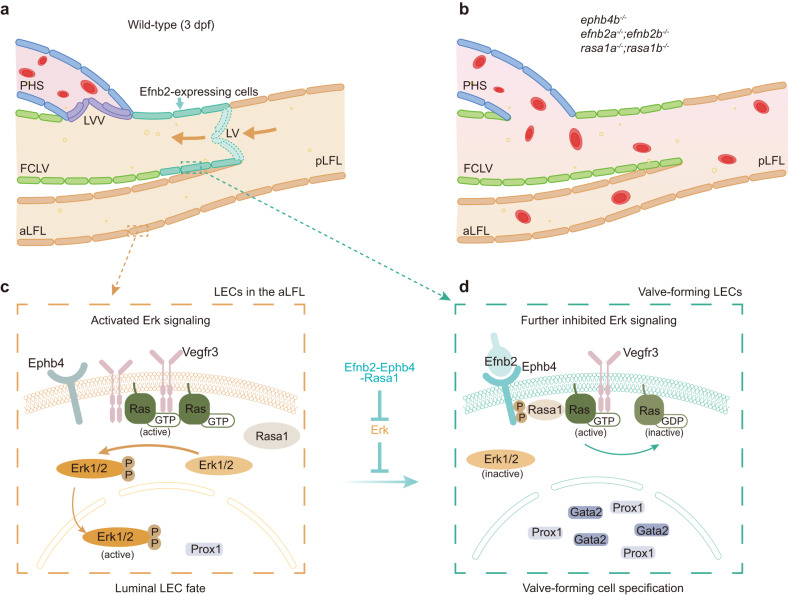
Inhibition of Erk activity in FCLV contributes to valve-forming LEC specification. **a** In wild-type embryos, FCLV-LVs and FCLV-PHS LVVs can be formed appropriately to ensure the unidirectional flow of lymphatic fluid and prevent the backflow of blood into the lymphatic circulation. **b** In the embryos with Efnb2-Ephb4-Rasa1 cassette mutations, FCLV-LVs and FCLV-PHS LVVs are defective, therefore leading to a kind of lymphatic blood-filling phenotype. **c** Vegfc-Vegfr3-mediated Erk activation in luminal LECs of the aLFL. **d** Efnb2-Ephb4-Rasa1 works in Erk inhibition to induce extremely high Prox1 expression in valve-forming cell specification. PHS primary head sinus, FCLV facial collecting lymphatic vessel, aLFL anterior part of lateral facial lymphatic vessel, pLFL posterior part of lateral facial lymphatic vessel, LV lymphatic valve, LVV lymphovenous valve. Venous endothelial cells (blue), the aLFL LECs with activated Erk signaling (orange), the FCLV LECs with repressed Erk signaling (green), Efnb2-expressing LECs (cyan), lymphatic valve to be formed (cyan with dashed lines), and FCLV-PHS LVV (purple) are indicated. The figure was created using Adobe Illustrator 2021

Then, we used MEK inhibitors to rescue valve defects in these two mutants. We applied five different MEK inhibitors, selumetinib, cobimetinib, trametinib, U0126-EtOH, and PD0325901, to *ephb4b*^−/−^ mutants from 2 to 4 dpf and used pericardial edema as the phenotypic readout (Fig. 7). MEK inhibition reduced the ratio of mutant embryos with pericardial edema (Fig. 7 and Supplementary Fig. 11c, d). In contrast, mTOR inhibitors rapamycin and BEZ235 did not rescue *ephb4b*^−/−^ mutant edema (Supplementary Fig. 11d). We checked the formation of LVs and LVVs in *ephb4b*^−/−^ mutants after treatment with 10 μM selumetinib and found that the *gata2a:EGFP*-labeled valve structure could be restored to a certain extent around 77 hpf (Supplementary Fig. 11e, f).

Rescue analyses in *efnb2a*^*−/−*^;*efnb2b*^*−/−*^ mutants showed different results. At 4 dpf, selumetinib treatment failed to reduce mutant edema. However, we discovered that more *gata2a:EGFP*-labeled valve-forming cells were present in *efnb2a*^*−/−*^;*efnb2b*^*−/−*^ mutant embryos given 100 μM selumetinib from 3 dpf, not only at the putative valve sites but also ectopically in the aLFL at 4 dpf (Fig. 7), even though these valve-forming cells were unable to form mature valve structures probably due to their poor organization. Thus, we propose that Efnb2, which is likewise critical for valve cell organization,^45,72^ enhances valve-forming cell specification together with Ephb4 by inhibiting Erk signaling.

Moreover, we found that Efnb2 expression is dynamic in FCLV LECs during lymphatic valve formation. At 60 hpf, FCLV LECs express a small amount of Efnb2 protein on their plasma membrane. Over time, its expression on the plasma membrane of FCLV LECs increases and becomes more constricted and elevated to a subset of FCLV LECs with Prox1a^hi^ expression, which indicates valve-forming LEC specification, at 77 hpf. Efnb2 remains on the plasma membrane of the valve cells at 5 dpf (Fig. 7). The dynamic expression of Efnb2 may be connected to its dual roles in regulating the specification of valve-forming cells and the organization of valve cells.

## Discussion

Previous genetic studies suggest that polarized expression of shear stress-responsive genes, including *gata2*, *prox1*, and *foxc2*, is required for the specification of valve-forming LECs during lymphatic valve development.^10,60^ An intriguing question is how *gata2* and *prox1* expression are particularly up-regulated in LECs that generate valve cells. Recent studies have shown that oscillatory shear stress can activate the canonical Wnt/β-catenin pathway to regulate valve specification.^10,73^ Unknown is whether another signaling mechanism is involved. Here, we discovered that, although Erk signaling must be engaged during the LEC formation, Erk inhibition is required for the specification of valve-forming cells. Firstly, prior to the initiation of the valve-forming cells, it is necessary to suppress Erk activity in the FCLV. Secondly, in addition to regulating the maturation and survival of valves, Efnb2-Ephb4-Rasa1 signaling is also required to suppress Erk activity in valve-forming cells to promote their specification in zebrafish (Fig. 8).

Activation of MAPK/Erk signaling by Vegfc-Vegfr3 is critical for the induction of LEC fate and thus promotes lymphatic vessel formation, whereas excessive Erk activation invariably leads to the lymphatic abnormalities observed in related lymphatic diseases.^24,26,74,75^ Loss of both *Spred1* and *Spred2* in mice, two genes that act to inhibit Vegfr3 signaling-activated Erk1/2, causes not only LEC overgrowth, but also embryonic edema and blood filling of lymphatic vessels.^76^ It has been reported that the abundance of *epsin1* and *epsin2* in collecting lymphatic vessels, but not in valve regions, mediates the internalization and degradation of Vegfr3, resulting in the termination of Vegfr3 signaling. Mice with LEC-specific deficiency of *epsin1* and *epsin2* had dilated lymphatic capillaries, immature lymphatic valves, and defective lymph drainage.^77^ Here, by using ERK-KTR sensor system, we discovered that Erk signaling is dynamically regulated during lymphatic valve development. In contrast to its beneficial function in lymphatic vessel development, suppression of Erk activity is essential for the initiation of valve-forming program, which might also be controlled by *spred1/2* or *epsin1/2* at this stage. This needs further investigation in zebrafish. However, because FCLV-LV cells are mostly derived from FCLV LECs, the developmental status of FCLV is also closely related to the formation of valve structures that follow. Therefore, the spatiotemporal regulation of Erk activity is critical for the generation of putative valve-forming cells.

Vegfc-Vegfr3 also activates PI3K-Akt signaling in LECs.^78^ PI3K-Akt signaling has been shown to stimulate de novo LV growth in mice, possibly by inactivating Foxo1, a key negative regulator, to inhibit the expression of valve-forming genes, such as *Gata2*, *Foxc2* and *Prox1*.^12^ Taking into account that PI3K-Akt inhibits Erk signaling via Akt1-dependent phosphorylation of Raf1 on Ser259 in LECs,^24,79^ Akt1-Raf1 crosstalk may be implicated in valve-forming LEC fate specification by controlling Erk1/2 activation. Combined with our findings, we conclude that the suppression of Erk activity is vital for LV development and may be achieved through the synergistic interaction of several signaling pathways. However, the molecular mechanism underlying the regulation of valve-forming genes by suppressed Erk signaling remains elusive and needs further investigation. One potential pathway implicated in this process is Notch signaling. Notch signaling has been shown to suppress Prox1 expression, ensuring the appropriate specification of LECs from venous ECs.^80^ Notch targets have been found to exhibit oscillatory patterns during somitogenesis and muscle stem cell differentiation,^81–83^ thereby regulating Erk activity oscillation.^63^ Additionally, studies in cancer have revealed a positive regulation of Notch signaling by the MAPK/Erk pathway.^84^ There might be a relationship between Notch pathway with Erk signaling in regulating valve formation.

We noticed that LVs and LVVs almost fail to form in *efnb2a*^*−/−*^;*efnb2b*^*−/−*^ double mutants, but partially form in *ephb4b*^*−/−*^ mutant larvae, suggesting that valve abnormalities are not the same in these mutants. Consistently, when using the MEK inhibitor to restore the valve formation in *efnb2a*^*−/−*^;*efnb2b*^*−/−*^ and *ephb4b*^*−/−*^ mutants, respectively, we found that the rescue effect in *efnb2a*^*−/−*^;*efnb2b*^*−/−*^ is inferior to that of *ephb4b*^*−/−*^ mutant. This phenomenon may be attributable to the fact that Efnb2 ligand activates other Eph receptors,^85–88^ including Epha4, Ephb1, and Ephb3, which may be expressed in FCLV LECs and hence influence valve development. Contrary to the observations made by Greysson-Wong et al.,^89^ we didn’t observe similar vascular defects in our *rasa1*^*−/−*^ mutants. We suspect there may be a leaky Rasa1b expression due to translation reinitiation after the premature termination codon, helping embryos avoid blood vascular defects.^90^ These ideas, however, need further investigation.

The EFNB2-EPHB4-RASA1 signaling axis is important for human vascular diseases, such as CM-AVM and VOGM, as revealed by a series of research involving whole exome sequencing.^39,40,91–93^ In addition to malformed blood vessels, lymphatic abnormalities can be observed in some CM-AVM and VOGM patients, and *EPHB4* mutations are found in lymphatic disorder CCLA patients.^35,37^ These findings indicate that EFNB2-EPHB4-RASA1 signaling regulates blood and lymphatic vessels in a complicated manner. Our study not only elucidates a possible mechanism underlying the lymphatic abnormalities in such diseases, but also establishes several zebrafish genetic disease models, including *ephb4b*^*−/−*^, *efnb2a*^*−/−*^;*efnb2b*^*−/−*^ and *rasa1a*^*−/−*^*;rasa1b*^*−/−*^, to better understand the pathogenesis of human lymphedema and evaluate potential therapeutic agents. Importantly, we found that treatment with MEK inhibitors significantly improves the lymphedema phenotype and restores valve formation in *ephb4b*^*−/−*^ or *rasa1a(-3)*^*−/−*^*;rasa1b*^*−/−*^ mutant larvae, providing a potential treatment strategy for valve-deficient disorders that currently lack specific molecular treatments.

## Materials and methods

### Zebrafish and maintenance

Zebrafish were raised and maintained with ethical approval from the Animal Care and Use Committee of Tsinghua University. The generation of mutants or transgenic lines is described below. Most lines were crossed with *Casper* mutant^94^ to obtain transparent embryos. Otherwise, 0.003% 1-phenyl-2-thiourea (PTU, Sigma, P7629) was used to inhibit pigment formation. *Tg(gata2a:EGFP)*^*la3*^, *Tg(gata1:DsRed)*^*sd2*^,^56^ *Tg(flk:EGFP)*^*s843*^,^95^ *Tg(fli1a:EGFP)*^*y1*^,^96^ and *Tg(flk:mCherry)*^97^ are described elsewhere. Abbreviations used in the text are listed in Supplementary Table 1.

### Transgenic fish generation

The *Tol2* transposon system was used to generate transgenic fish. For *Tg(lyve1b:TopzaYFP)*^*tsu47tg*^, *Tg(lyve1b:DsRed2)*^*tsu48tg*^, and *Tg(lyve1b:kaede)*^*tsu52tg*^, the 5.2 kb *lyve1b* promoter was used as previously reported.^55^ For *Tg(epb41b:mCherry)*^*tsu49tg*^, a region containing 3738 bp upstream of *epb41b* start codon was amplified. For *Tg(gata2aECE:ERKKTR)* ^*tsu50tg*^, *Tg(gata2aECE:nls-mCherry)* ^*tsu51tg*^, and *Tg(ef1α:ERKKTR)*^*tsu53tg*^, *gata2a* endothelial core element (*gata2aECE*) and ERK-KTR biosensor element were synthesized as reported previously.^13,64^ In our case, 20 pg *Tol2*-flanked transgenic construct DNA and 200 pg *Tol2* transposase mRNA were co-injected into 1-cell embryos with a Tuebingen background. The selected founder was then crossed with a wild-type fish, generating a population of heterozygous and wild-type offspring (F1). Please see Supplementary Table 3 for the primer information.

### Generation of knockout lines and genotyping

The CRISPR/Cas9 system was used to make knockout lines. The Cas9 mRNA and sgRNAs were synthesized using the mMESSAGE mMACHINE T7 Transcription kit (ThermoFisher, AM1344). A total of 200 pg Cas9 mRNA or 1–3 fmol Cas9 protein (NEB, M0646T) with 100–400 pg corresponding sgRNAs were co-injected into Tuebingen embryos at the 1-cell stage. The founder fish and F1 offsprings with mutations were identified by sequencing. For genotyping, embryos were lysed in 30–50 μl 50 mM NaOH per embryo and then neutralized with 3–5 μl 1 M Tris-HCl (pH 8.0). After PCR with the relevant primer sets, the PCR products were either directly run through a high concentration agarose gel (4%) or cut by specific restriction enzymes. *erk1*^−/−^ (*erk1*^*tsu45*^), *erk2*^−/−^ (*erk2*^*tsu46*^), *ephb4a*^−/−^ (*ephb4a*^*tsu37*^), *efnb2a*^−/−^ (*efnb2a*^*tsu41*^), *efnb2b*^−/−^ (*efnb2b*^*tsu42*^), *rasa1a*^−/−^ (*rasa1a*^*tsu38*^), *rasa1a(-3)*^−/−^(*rasa1a*^*tsu40*^), and *rasa1b*^−/−^ (*rasa1b*^*tsu39*^) mutants were generated. Please see Supplementary Table 3 for specific DNA oligos used as sgRNA templates, and the primers for mutant identification, and see Supplementary Table 2 for additional allele information.

### Whole mount immunostaining

Embryos were fixed with 4% paraformaldehyde (Sigma, P6148) in PBS for 1–2 days at 4 °C, and then transferred to 100% methanol and stored at −20 °C for at least 1 day. Before immunostaining, embryos were rehydrated in PBS and their tails were cut off and lysed in 50 mM NaOH for genotyping. For Prox1a detection, embryos were treated with 1 mM EDTA (pH 8.0) at 90 °C for 10 min, and then blocked in 2% BSA for 2 h. For p-Erk and Efnb2a detection, embryos were treated with 10 ng/μl proteinase K (Amresco, 0706-100MG) at 28.5 °C for 60 min. Rabbit anti-phospho-Erk (Thr202/Tyr204) (1/200; CST, 4370), rabbit anti-Prox1 (1/200; GeneTex, GTX128354), chicken anti-GFP (1/200; Abcam, ab13970), goat anti Efnb2a (1/500; R&D, AF1088), mouse anti-GFP (1/100; EASYBIO, BE2003), and mouse anti-mCherry (1/100; EASYBIO, BE2026) were used for primary incubation. Goat anti-chicken IgY-Alexa Fluor 488 (1/400; Abcam, ab150169), goat anti-rabbit IgG-Alexa Fluor 647 (1/400; Jackson, 111-605-003), goat anti-mouse IgG-TRITC (1/400; Jackson, 115-545-003), donkey anti-goat IgG-647 (1/400; Abcam, ab150131), donkey anti-rabbit IgG-TRITC (1/400; Jackson, 711-025-152), and donkey anti-mouse IgG-488 (1/400; Abcam, ab150105) were used for secondary incubation. The embryos were washed several times with 0.2% Trixton X-100 in PBS before being merged with 2% propyl gallate (Sigma, P3130) antifade solution and mounted on glass slides with home-made tapes.

### Transmission electron microscopy

Embryos were fixed with 2.5% glutaraldehyde in 0.1 M PB buffer at 4 °C and dehydrated in a gradual ethanol series. Embryos were then infiltrated with and embedded in SPON12 resin at 60 °C for 2 days for polymerization. Samples were cut on an Ultracut microtome. Ultrathin sections were collected every 1–2 μm when lymphatic vessels were observed in transmission electron microscope and picked up with Formvar-coated copper grids (100 mesh). After double staining with uranyl acetate and lead citrate, the sections were examined by a transmission electron microscope H-7650B with an acceleration voltage of 80 kV.

### Whole mount in situ hybridization (WISH)

Embryos were fixed for 1–2 days at 4 °C with 4% paraformaldehyde (Sigma, P6148) in PBS, and then transferred to 100% methanol and stored at −20 °C for at least 1 day. Whole mount in situ hybridization was carried out following the standard protocol. A DIG-labeled antisense RNA probe for *erk1* was synthesized using Roche DIG RNA Labeling Mix (11277073910). Refer to Supplementary Table 3 for the primers.

### Small molecule treatment

The embryos at sphere stages were incubated in 6-well plates with 3 ml 100 μM selumetinib till 24 hpf. For time-lapse imaging, embryos were dechorionated and incubated with drugs from sphere to shield stage, then embedded in 0.8% low-melting agarose gel covered by drug solution. For MEK inhibition in *ephb4b*^*−/−*^, embryos at 2 dpf were transferred into 6-well plates with 30 embryos per well and 3 ml of 25% Holtfreter’s water with small molecules or DMSO. The embryos were raised to 4 dpf at 30.5 °C to observe pericardial edema. Rapamycin (Solaribio, R8140-25), BEZ235 (Topscience, T2235), and MEK inhibitors selumetinib (Topscience, T6218), cobimetinib (Topscience, T3623), trametinib (Topscience, T2125), U0126-EtOH (Topscience, T6223), and PD0325901 (Selleck, S1036) were used. For *rasa1*^−/−^ mutants and wild-type embryos, 10-100 μM selumetinib in 100% Holtfreter’s water was used.

### Imaging and image processing

Embryos or larvae were anesthetized with 0.2 mg/ml Tricaine (Sigma, A5040) and embedded in 1% or 0.8% low melting agarose/Holtfreter’s water in 35-mm glass bottom culture dishes. Imaging was carried out on a Perkin Elmer Spinning Disk confocal or Dragonfly Spinning Disk confocal microscope (Andor) using a 20x objective. For time-lapse imaging of Erk reporter fish, embryos were embedded in 0.4% low melting agarose and imaged at 40 min intervals using a 40x oil objective lens on a 30.5 °C heated stage. Time-lapse imaging of embryos with *Tg(ef1α-ERKKTR)* from shield stages were carried on a 20× objective lens of an FV3000 with a 10-min interval on a 30.5 °C heated stage. For immunostaining, a 40× oil objective was used. Blood and lymph autofluorescence can be detected in the λex 561 nm channel. Embryos with unknown genotypes were imaged first, and then genotyped (see Table S1). As for adult fish caudal heart, fish was first fixed in 10 ml 4% PFA for 1 h at room temperature. Then washed with PBS for several times. The skin and muscle near the caudal heart were removed using forceps. Imaging was performed on the remaining tails. For photoconversion, the *Tg(lyve1b:Kaede)* larva were mounted in 1% low melting point agarose and observed under an FV3000 inverted confocal microscope (Olympus) with 20× objective. The focused LECs were irradiated by the 405 nm laser for 3 s. Imaris 9.0.1 was used for image processing. Single plane or 3D images were generated by Snapshot and processed by Adobe Photoshop 2020. Movies were generated using Imaris 9.0.1, imported into Adobe Premiere Pro 2020 for labeling, and finally exported as .mov files.

### Lymphangiography and angiography

In tail lymphatic vessel lymphangiography, 5 mg/ml 2000 kDa Dextran-Rhodamine (D7139, Invitrogen) was injected directly into the tail lymphatics using an oil injector under fluorescence stereomicroscopy. The video was taken immediately after injection. For lymphangiography in the trunk, 1-2 nl 10 mg/ml 2000 kDa Dextran-Rhodamine was directly injected into the trunk. After 10-30 min, the larvae were embedded for confocal imaging. Adult retro-orbital injection was performed as described before^98^ with modifications. Injections could be made into the tissue behind the eyes as well as the retro-orbital venous sinus. The surrounding lymphatic vessels could take up the Dextran-fluorescein and finally entered the blood circulation. A 10 μl hamilton syringe was used, and the tip was hand honed to be shorter and smoother. Adult fish was anesthetized with 0.2 mg/ml tricaine and positioned dorsal up, head right. The needle was inserted 1-2 mm deep at a 45-degree angle to the fish at seven o’clock position. 3 μl 5 mg/ml 2000 kDa Dextran-fluorescein (D7137, Invitrogen) was injected. For the angiography at 4 dpf, 1-2 nl 5 mg/ml 2000 kDa Dextran-Rhodamine was injected into the CCV. Dextran-Rhodamine would be observed circulating in the bloodstream immediately after injection. The successful injection ratio was around 50%.

### Embryonic Western-blot

4 dpf embryos were collected and anesthetization with Tricaine after being treated with DMSO or 100 μM selumetinib. Yolk was removed by blowing and sucking using a 200 μl pipette. TNE (0.15 M NaCl, 5 mM EDTA, 10 mM Tris-HCl and 1% Triton X-100) plus protease inhibitor (Roche, 04693132001) and phosphatase inhibitor NaF and Na_3_VO_4_ was used to lyse the embryos, which were then homogenized using a TGrinder (TIANGEN, OSE-Y50). After boiling in SDS loading buffer, the samples were centrifuged at 12,000 × *g* for 5 min. The supernatant was used for a typical western blot with a 4-20 % precast gel (EASYBIO, BE6931). Mouse Erk 1/2 antibody (1/2000; Santa Cruz, 514302), rabbit p-Erk1/2 antibody (1/5000; CST, 4370), and rabbit Actb antibody (1/100000; Abclonal, AC026) were used for primary incubation.

### Data processing and statistical analysis

To quantify Erk activity in FLV, we used two methods. The first method is to measure the cytoplasm-to-nuclei total intensity ratio. The 3D images were rotated and processed into maximum intensity projection in Imaris 9.0.1. Only cells with obvious EGFP and NLS-mCherry expression were chosen for analysis in Fiji. The nucleus was selected by NLS-mCherry fluorescence with thresholding. Image Calculate-Subtract function was used to generate the ERKKTR-EGFP fluorescence in cytosol. Single cell was marked manually as region of interest (ROI). Total fluorescence intensities of the whole cell and cytosol were calculated with the same thresholding using one ROI. Finally, the cytoplasm-to-nuclei ratio was calculated to represent Erk activity. Same method for calculating Erk activity in whole FCLV and aLFL, as well as the C/N ratio after selumetinib treatment.

The second method is to measure the mean intensity ratio of cytoplasm (define a ring from nuclei) to nuclei. The procedure was carried out as previously described.^99,100^ The images were first deconvoluted by Huygens and removed unnecessary parts before converted to 2-D by maximum intensity projections in Imaris. Embryos with low KTR expression in FCLV were not suited for calculation because they produced falsely high Erk activity. The nuclei were identified using the Bernsen Auto Local Threshold method, and defined as cells after being dilated twice (two pixels). Fluorescence intensity was measured with Huang auto threshold values.

To quantify Erk activity at the margin region in *Tg(ef1a:ERKKTR)* embryos, the z-stacked images were first processed into maximum intensity projections using Imaris 9.0.1. The margin region was cropped for analysis in Fiji. First, images were subjected to Subtract Background for signal intensity analysis. In DMSO-treated group, the images were first smoothened and Gaussian blurred with sigma = 1, and then nuclei were found using the Triangle threshold method with low value (from 2 to 7 or 10) because the nuclei were dimmer. The nuclei were bright in selumetinib-treated group, and were identified using an auto Triangle threshold method (argument, ignore black). The nucleus masks were dilated twice (2 pixels) to define cell masks. Nucleus and cell regions and mean intensities were measured and the C/N ratio of mean intensity was calculated in Excel.

When analyzing the ERKKTR-EGFP fluorescence in the nucleus in time-lapse imaging from 72 to 78 hpf, we compared the ratio of nuclear total intensity changes in the nuclei.^101^ Nucleus equivalent surfaces were created based on the expression of nls-mCherry using Imaris 9.8.0 with a smooth factor 0.7 and thresholding around 112. Surfaces objects with low ERKKTR expression were deleted. Unsuitable Surfaces with multiple nuclei were manually removed or separated. Nuclei tracking was corrected thereafter. These Surfaces were then used to calculate the ERKKTR-EGFP fluorescence intensity in the nucleus. To depict the Erk activity, intensities were normalized by the first value and then scaled by log10.

Cells with Prox1a^hi^ expression and gata2a:EGFP expression were considered as valve cells. When gata2a:EGFP was not detected, only cells with Prox1a^hi^ expression clustered in the FCLV were considered as valve-forming cells. The relative intensity of p-Erk1/2 in immunofluorescence experiments was determined by selecting three or more regions of FCLV in Imaris 9.0.1 and measuring the mean signal intensity of p-Erk1/2 and lyve1b:DsRed2. The relative p-Erk1/2 intensity was defined as the ratio of p-Erk1/2 intensity to lyve1b:DsRed2 intensity. Statistical analyses were performed in GraphPad Prism 8.2.1 and the mean ± SD were calculated. The unpaired and paired student’s *t*-test, chi-squared test, and Fisher’s exact test were used to evaluate significance. The sample size (n) and *P* value for each experimental group were described in corresponding figures and figure legends.

### Supplementary information

Supplementary files

Supplementary Movie 1

Supplementary Movie 2

Supplementary Movie 3

Supplementary Movie 4

Supplementary Movie 5

Supplementary Movie 6

Supplementary Movie 7

Supplementary Movie 8

Supplementary Movie 9

Supplementary Movie 10

Supplementary Movie 11

Supplementary Movie 12

Supplementary Movie 13

Supplementary Movie 14

## Data Availability

All data are available in the article or the online supplementary materials or upon reasonable request to corresponding authors.
