# Supplementary material for: Temporospatial inhibition of Erk signaling is required for lymphatic valve formation
Source: Signal Transduct Target Ther. 2023 Sep 11;8:342. doi: 10.1038/s41392-023-01571-9 (PMC10493226; doi:10.1038/s41392-023-01571-9)
Supplement: Supplementary file 1 — Supplementary files [file 41392_2023_1571_MOESM1_ESM.docx]

Supplementary Materials for

Temporospatial inhibition of Erk signaling is required for lymphatic valve formation

Yaping Meng,^1^† Tong Lv,^1^† Junfeng Zhang,^2^ Weimin Shen,^1^ Lifang Li,^1^ Yaqi Li,^1^ Xin Liu,^1^ Xing Lei,^3^ Xuguang Lin,^1^ Hanfang Xu,^3^ Anming Meng,^1,2,^* Shunji Jia^1,3,^*

Correspondence to: [jiasj@genetics.ac.cn](mailto:jiasj@genetics.ac.cn) (S.J.); [mengam@mail.tsinghua.edu.cn](mailto:mengam@mail.tsinghua.edu.cn) (A.M.)

**This PDF file includes:**

Figures. S1 to S11

Captions for Movies S1 to S14

Tables S1 to S3

Reference

**Other Supplementary Materials for this manuscript include the following:**

Movies S1 to S14


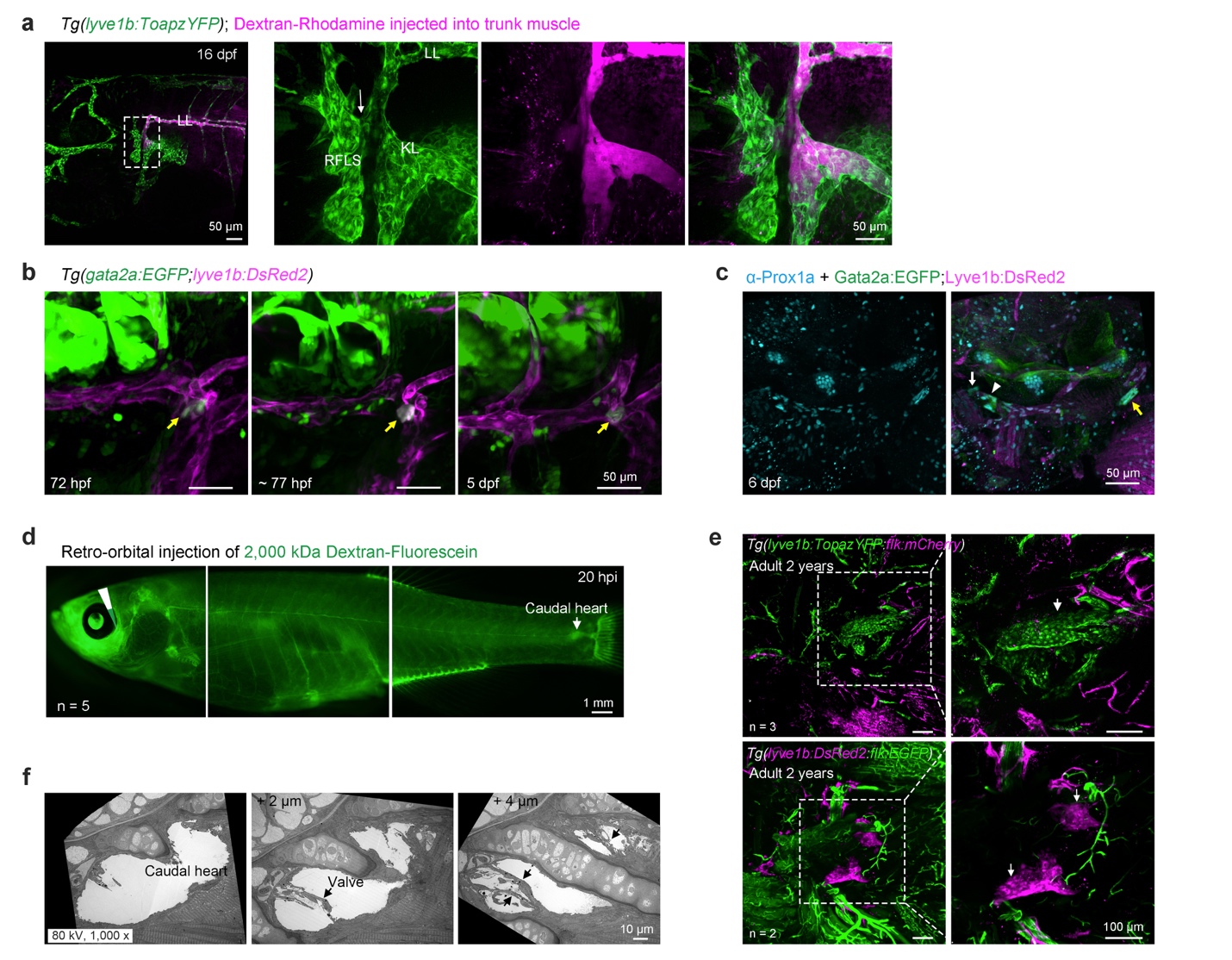


Figure. S1. RFLS in the head and caudal heart in the tail.

**a,** Lymphangiography at 16 dpf. Dextran-Rhodamine was injected into the trunk muscle and absorbed by lateral lymphatics (LL), which then flowed into RFLS, indicating a link between LL with RFLS. **b**, Uncropped images of RFLS-CCV LVV structures presented in Figure 1b. **c**, Uncropped images of Prox1a immunostaining at 6 dpf related to Figure 1c. **d,** Retro-orbital injection of 2,000 kDa Dextran-Fluorescein in adult fish. Dextran-fluorescein could be absorbed by the surrounding lymphatic vessels and further entered the blood circulation. Arrow indicated the caudal heart. **e**, Caudal heart (arrows) in adult fish could only be labeled by *lyve1b:TopazYFP/lyve1b:DsRed2.* **f,** TEM of the caudal heart. Arrows indicated the valve leaflet in caudal heart. All images are anterior to the left, dorsal upward.


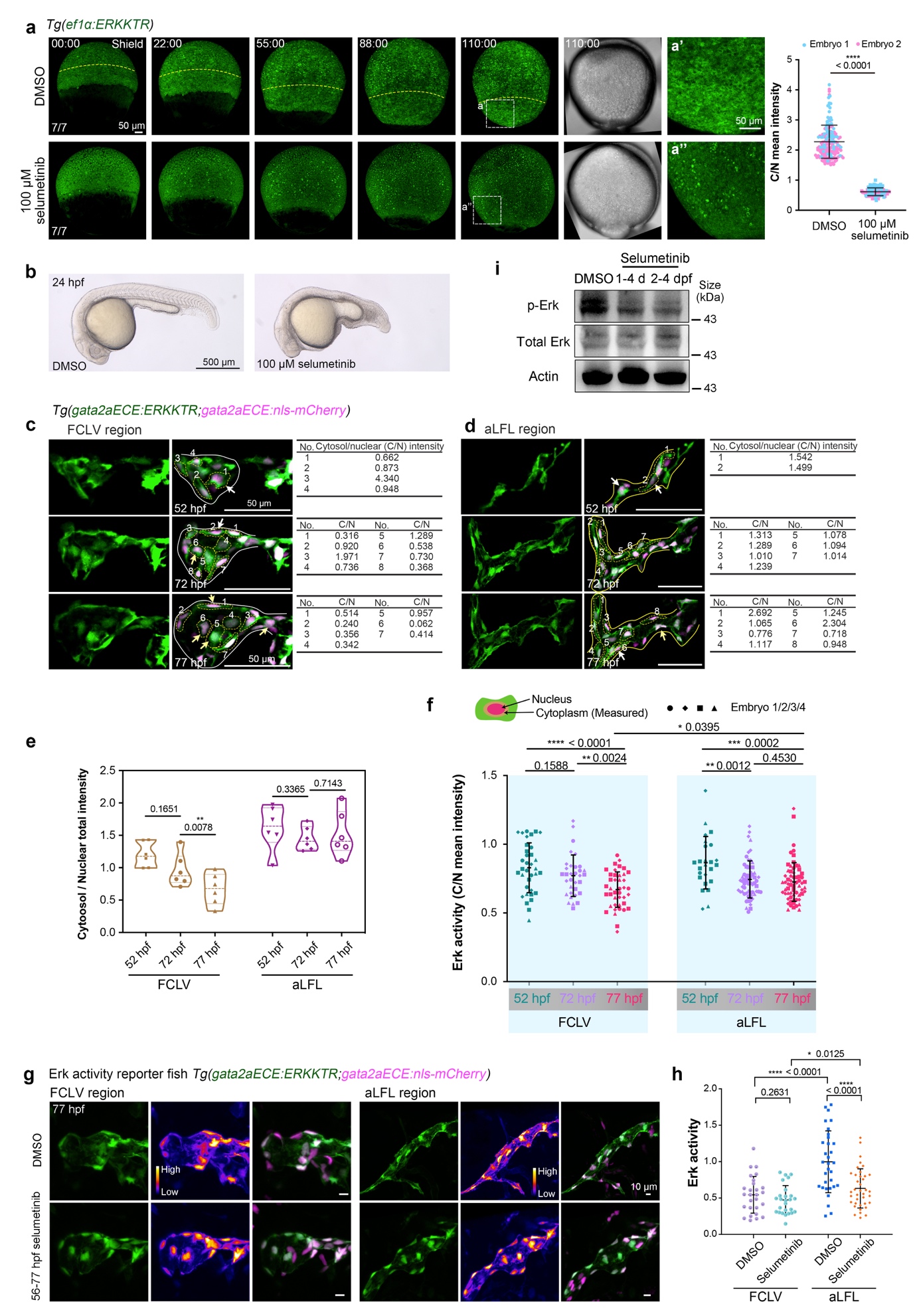


Figure. S2. The verification and examination of Erk activity in ERKKTR reporter line.

**a**, Erk activity detected by ERK-KTR biosensor in *Tg(ef1α:ERKKTR)* fish during gastrulation. Selumetinib treatment from the sphere stage disrupted the specific activation of Erk signaling near the embryonic margin. Erk activity at the marginal region was calculated and statistical analysis was conducted based on data from two embryos. Unpaired two-tailed *t* test (*N* = 3 independent experiments; n = 8 for DMSO, n = 8 for selumetinib). Scale bars, 50 μm. All images are animal pole to the top, dorsal to the right. **b**, Embryos with selumetinib treatment in a displayed anterior-posterior patterning defect at 24 hpf. Scale bar, 500 μm. **c**, The Erk activity represented by cytosol/nuclear fluorescence ratio in FCLV LECs in Fig. 2d are shown. Scale bars, 50 μm. **d**, Erk activity in aLFL LECs in Fig. 2d are shown. Scale bars, 50 μm. **e**, Erk activity in whole region of FCLV and aLFL. Paired two-tailed *t* test (n = 6). **f**, Erk activity in FCLV and aLFL was measured by defining a ring from the nuclei as cytoplasm. Unpaired two-tailed *t* test (n = 4). **g**, Erk activity in FCLV and aLFL after MEK inhibitor selumetinib treatment. Scale bars, 10 μm. **h**, Quantification of Erk activity in individual cells after MEK inhibition. Unpaired two-tailed *t* test (n = 6 for DMSO, n = 7 for 100 μM selumetinib). **i**, p-Erk protein level decreased after 100 μM selumetinib, detected by western blot.


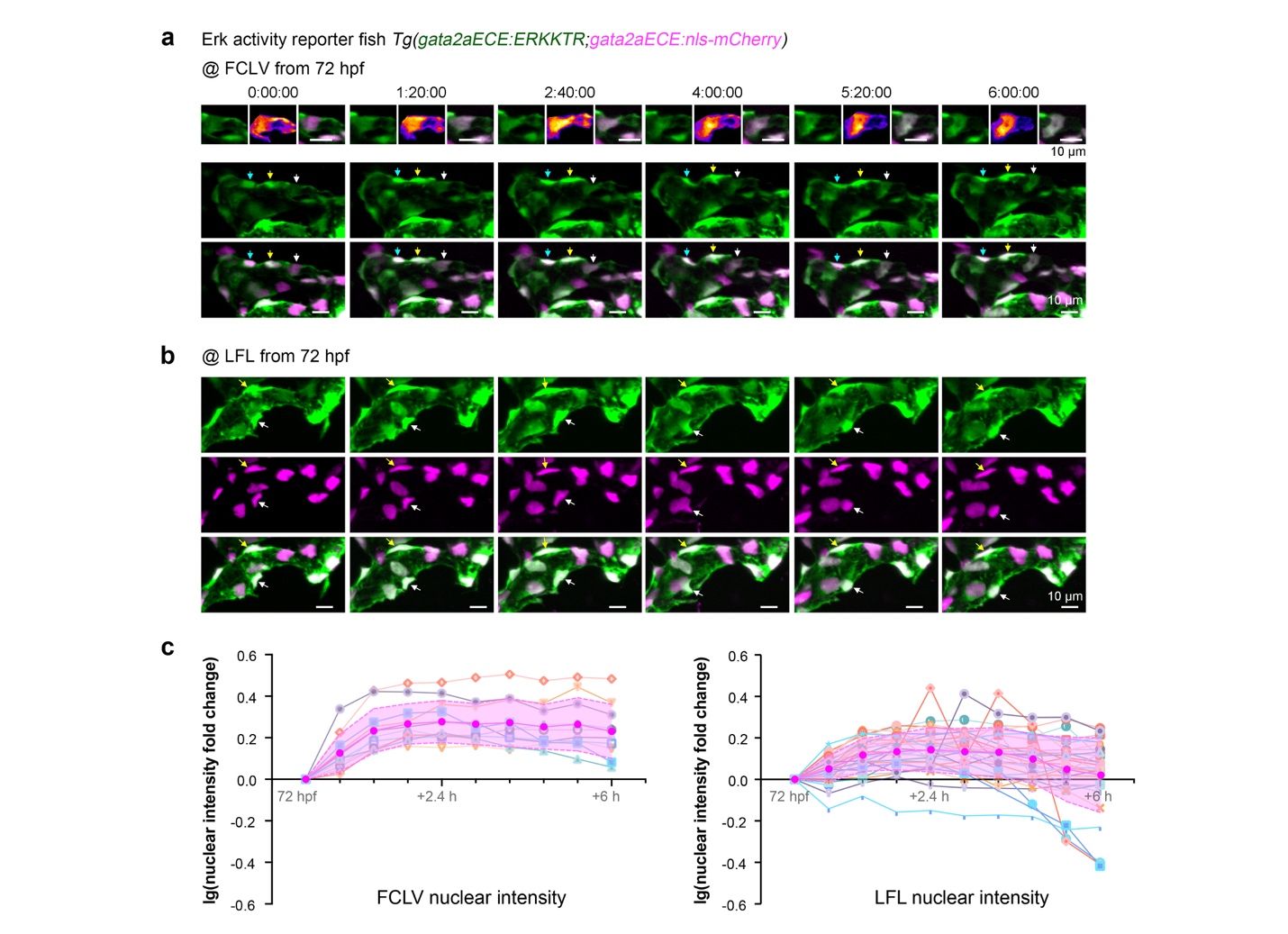


Figure. S3. A decrease of Erk activity in FCLV LECs from 72 to 78 hpf.

**a**,**b**, Time-lapse imaging of Erk reporter fish from 72 hpf. Arrows indicated the individual cells. The top panel showed a typical cell with decreased Erk activity during development, with whole cell Fire LUT (Fiji) in the middle. **c**, ERKKTR fluorescence changes in LEC nucleus in FCLV and aLFL from 72 to 78 hpf. Each line represented a single cell. The purple lines represented the mean and standard deviations. Cells from two embryos were shown and a total of n = 6 embryos from two independent experiments were examined.


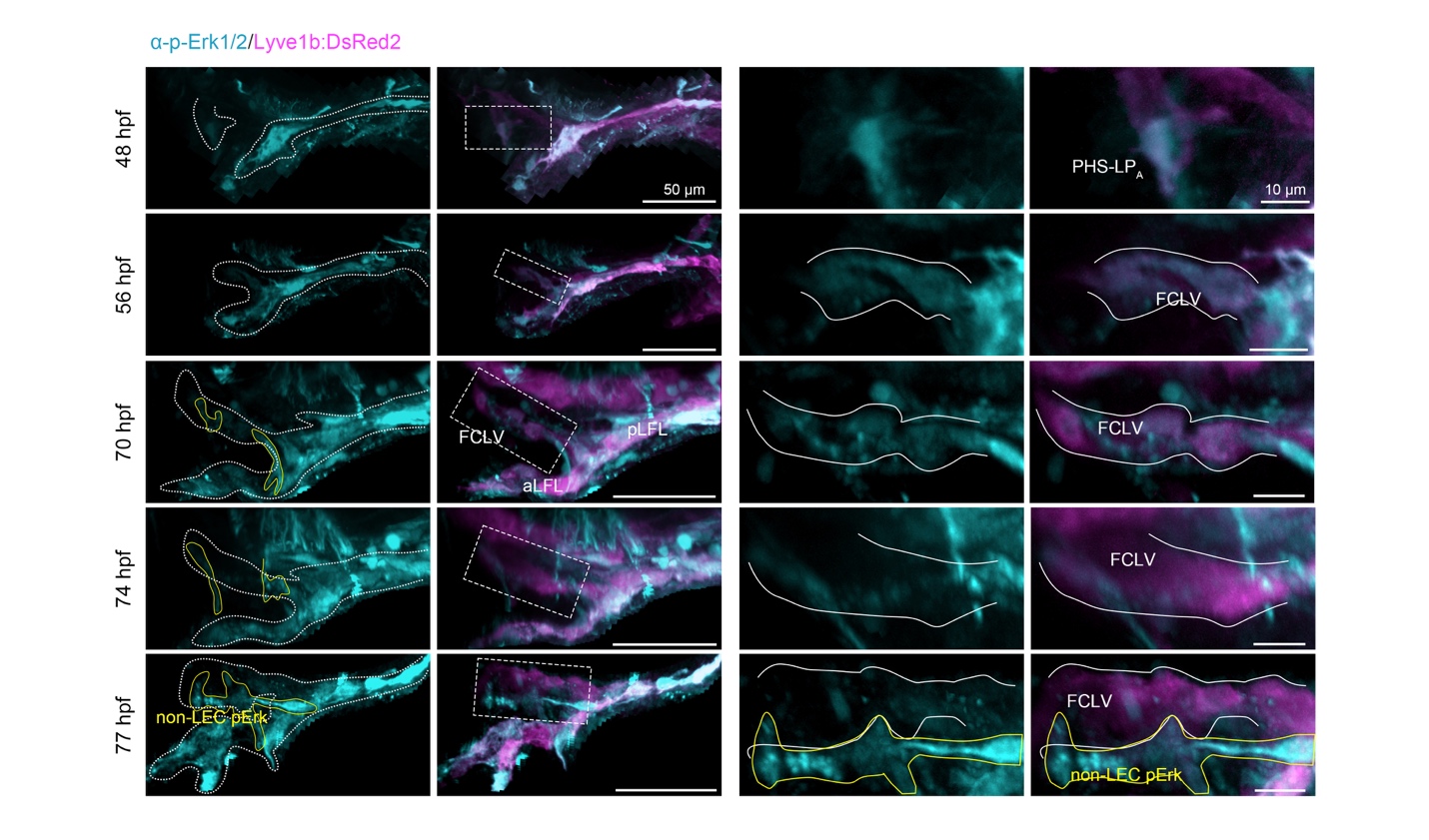


Figure. S4. Whole mount p-Erk1/2 immunostaining revealed a decrease of Erk signal in FCLV.

Whole mount p-Erk1/2 immunostaining of FLVs from 48 hpf to 77 hpf. At 48 hpf, FCLV progenitors (PHS-LP_A_) is shown. The right two panels display the boxed areas. The FCLV is denoted by white lines. Non-lymphatic tissues with Erk signal (non-LEC pErk) are shown by yellow lines. Scale bars, left two panels, 50 μm; right two panels, 10 μm.


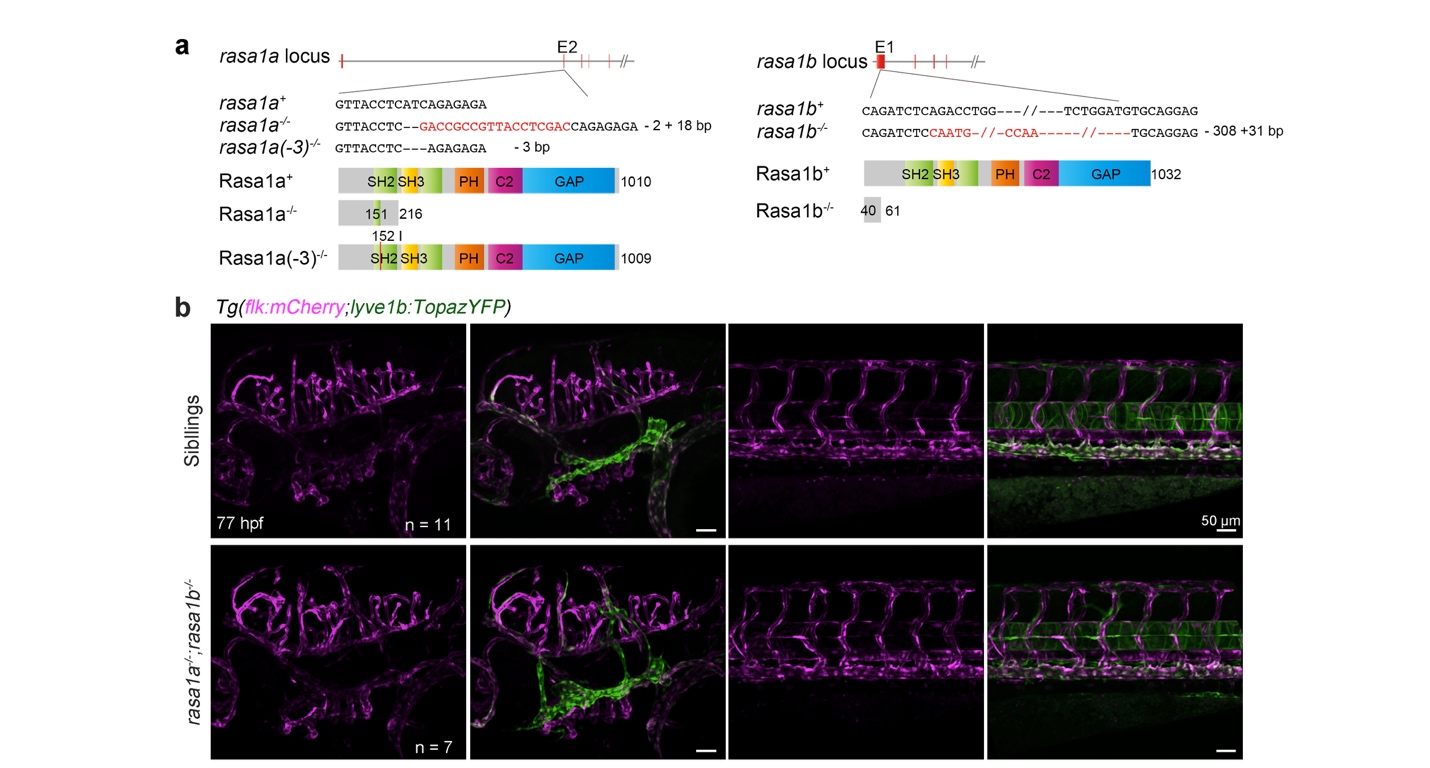


Figure. S5. *rasa1a^-/-^;rasa1b^-/-^* mutants showed an overgrowth of lymphatic vessels.

**a**, Generation of the *rasa1a* and *rasa1b* mutants. The protein length of mutant Rasa1a is 216 aa. The amino acid sequence changes after 151 aa. The Rasa1a (-3)^-/-^ mutant bears a 3-bp deletion with loss of the isoleucine (I) at 152 aa. The protein length of mutant Rasa1b is 61 aa. The amino acid sequence changes after 40 aa. **b**, In both the head and trunk areas of *rasa1a^-/-^;rasa1b^-/-^* double mutants, blood vessels labeled by *flk:mCherry* can be formed appropriately, while the head region shows an overgrowth of the lymphatic vessels labeled by *lyve1b:TopazYFP*. Scale bars, 50 μm. All images are anterior to the left, dorsal upward.


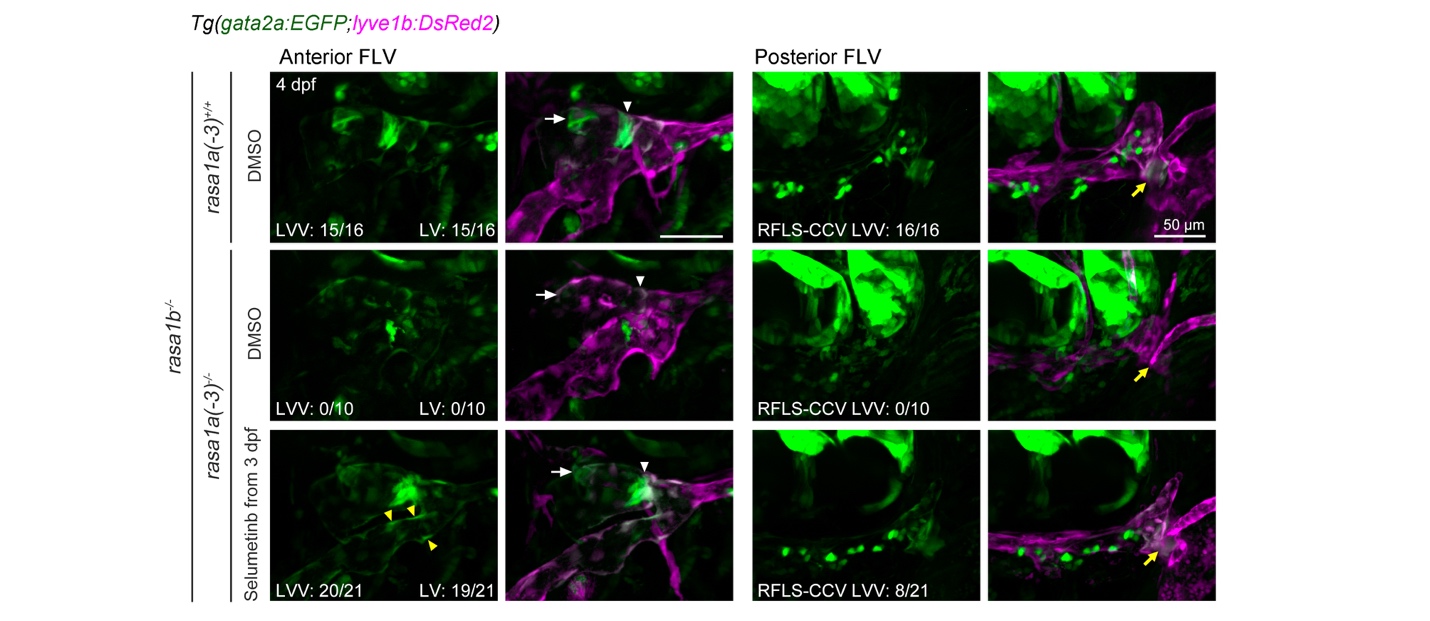


Figure. S6. MEK inhibition from 3 to 4 dpf could restore valve structure in *rasa1a(-3)*^-/-^;*rasa1b*^-/-^.

Selumetinib treatment from 3-4 dpf restored the valve structure in *rasa1a(-3)*^-/-^;*rasa1b*^-/-^ labeled with *gata2a:EGFP* and also induced ectopic *gata2a:EGFP* expression in the aLFL. Arrowheads, LVs; arrows, FCLV-PHS LVVs; yellow arrows, RFLS-CCV LVVs; yellow arrowheads, ectopic *gata2a:EGFP* positive cells. The numbers of embryos with indicated phenotype are shown. Scale bars, 50 μm. All images are anterior to the left, dorsal upward.


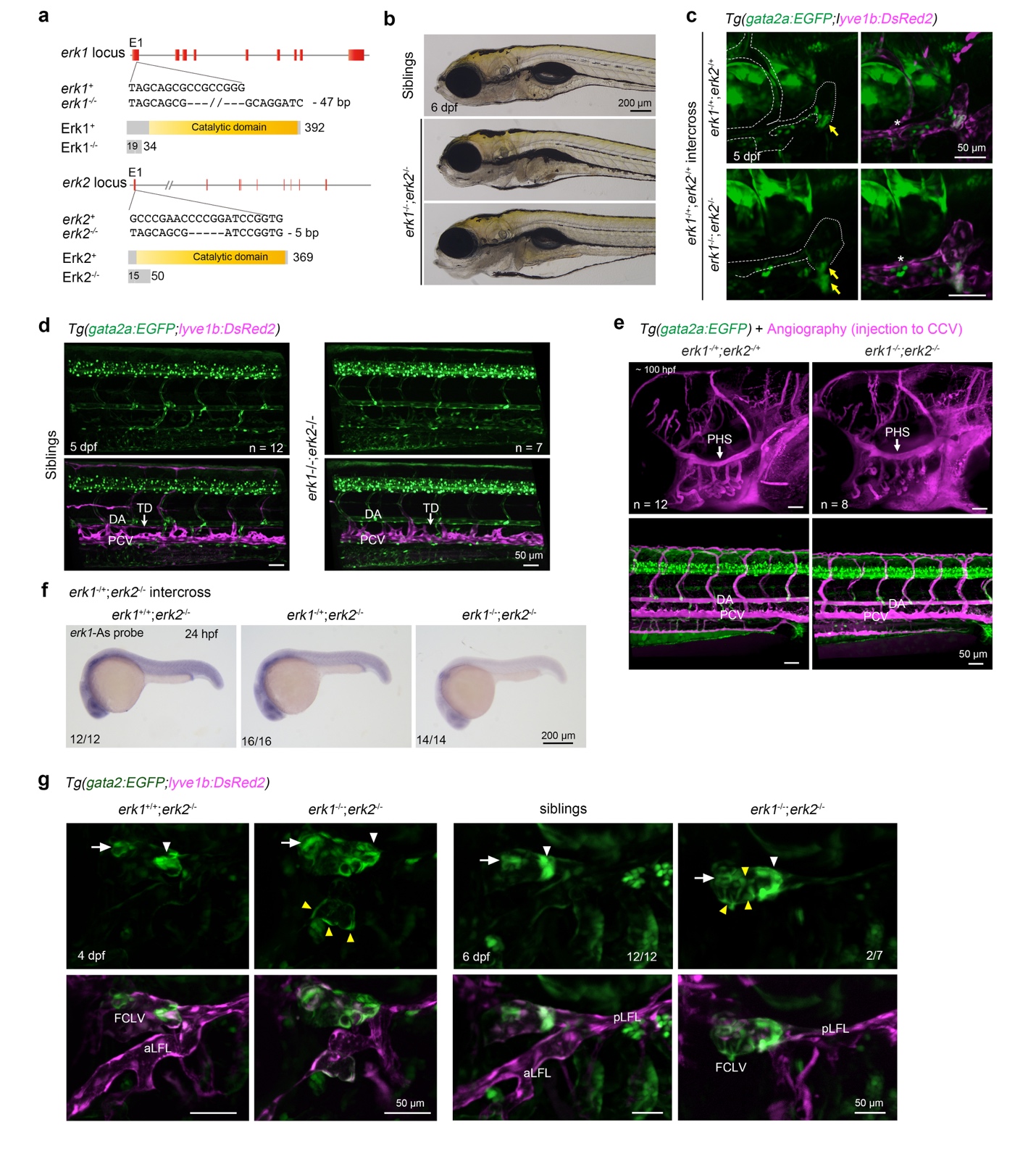


Figure. S7. Normal blood vasculature, but defective lymphatic vasculature, and ectopic *gata2a:EGFP* in facial lymphatic vessels in *erk1^-/-^;erk2^-/-^* mutants.

**a**, Generation of the *erk1* and *erk2* mutants using CRISPR/Cas9 technology with a target site at exon 1. The *erk1*^-/-^ mutant bears a 47-bp deletion with a protein length of 34 aa. The amino acid sequence changes after 19 aa. The *erk2*^-/-^ mutant bears a 5-bp deletion with a protein length of 50 aa. The amino acid sequence changes after 15 aa. **b**, *erk1^-/-^;erk2^-/-^* double mutants develop edema at 6 dpf. Scale bar, 200 μm. **c**, Loss of the otolithic lymphatic vessel (OLV, asterisks) and overgrowth of the RFLS-PHS LVV (yellow arrows) in *erk1*^-/-^;*erk2*^-/-^ mutants. Scale bars, 50 μm. **d**, Absence of the thoracic duct and appropriate development of trunk blood vessels in *erk1*^-/-^;*erk2*^-/-^ mutants. The thoracic duct and posterior cardinal vein are labeled by *lyve1b:DsRed2*, while the dorsal aorta is labeled by *gata2a:EGFP.* DA, dorsal aorta; PCV, posterior cardinal vein; TD, thoracic duct. n, the number of embryos observed. Scale bars, 50 μm. **e**, Angiography revealed that blood circulation was normal in *erk1*^-/-^;*erk2*^-/-^ mutants. Scale bars, 50 μm. **f**. *erk1* whole mount *in situ* hybridization at 24 hpf in *erk1^+/+^;erk2^-/-^, erk1^-/+^;erk2^-/-^*, and *erk1^-/-^;erk2^-/-^* embryos obtained by intercrossing *erk1^-/+^;erk2^-/-^* fish. Scale bar, 200 μm. **g**, Ectopic *gata2a:EGFP* expression (yellow arrowheads) in the FCLV and aLFL region of *erk1^-/-^;erk2^-/-^* mutant embryos at 4 dpf and 6 dpf. Scale bars, 50 μm. All images are anterior to the left, dorsal upward.


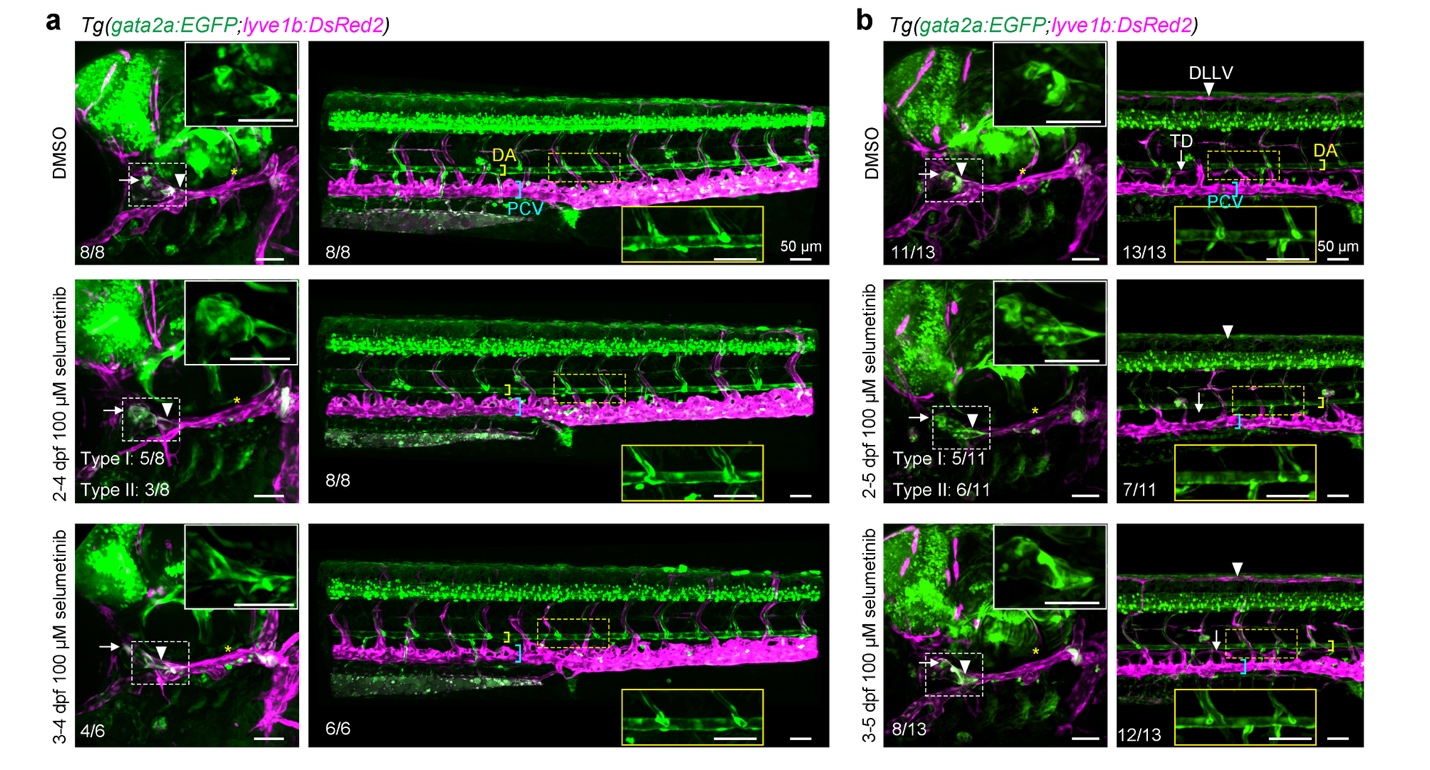


Figure. S8. MEK inhibition could not induce ectopic *gata2a:EGFP* expression in the trunk blood or lymphatic vasculature.

**a**, MEK inhibition by selumetinib from 2 or 3 dpf to 4 dpf could induce valve hyperplasia in FLV, but not ectopic *gata2a:EGFP* expression in the trunk vessels. Arrows, FCLV-PHS LVV; arrowheads, lymphatic valves. Enlarged images of the boxed regions were inserted. **b**, MEK inhibition by selumetinib from 2 or 3 dpf to 5 dpf could not induce ectopic *gata2a:EGFP* expression or valves in the trunk. Arrows, TD; Arrowheads, DLLV. Scale bars, 50 μm.


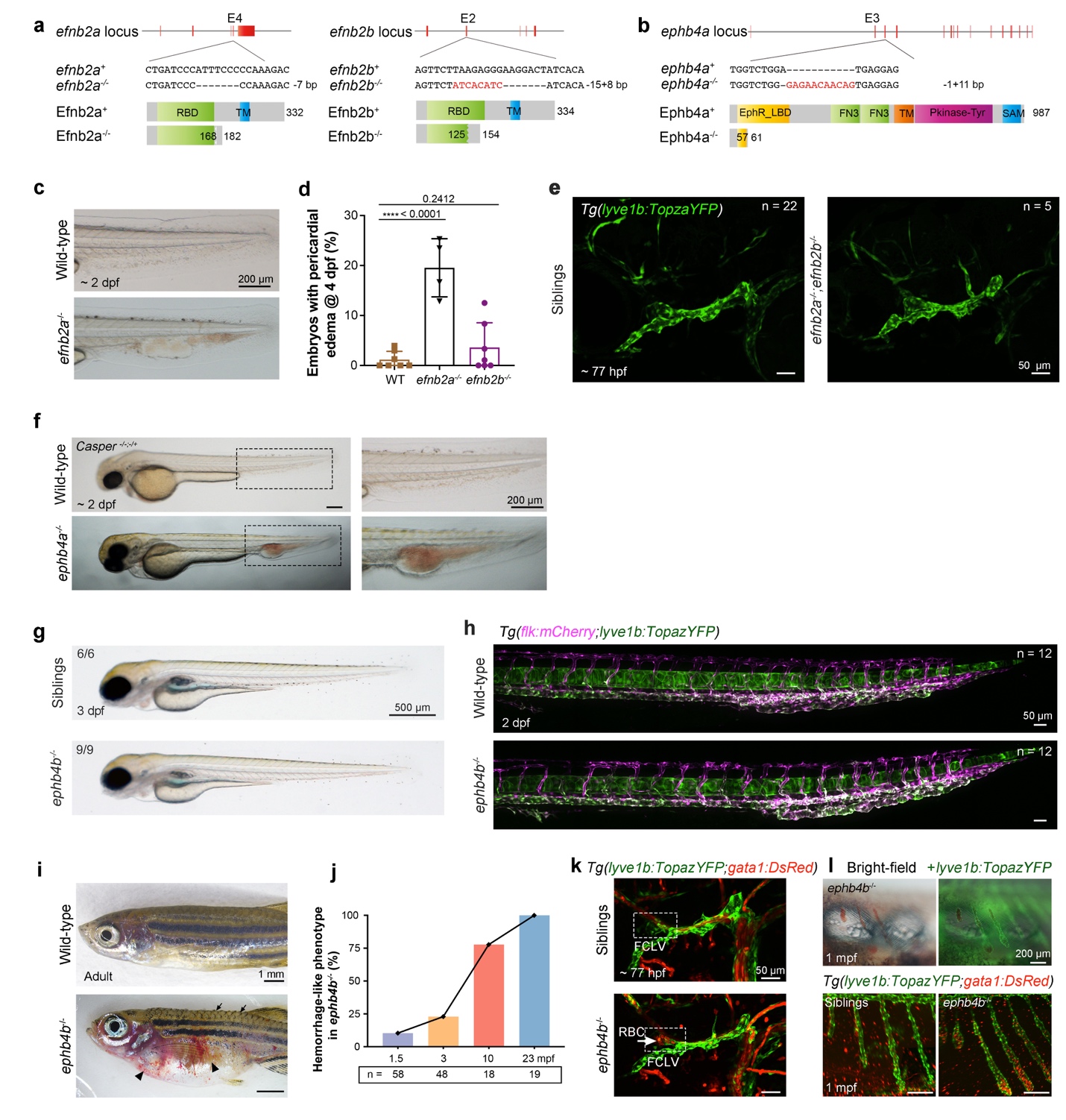


Figure. S9. Blood and lymphatic vasculature phenotypes in *efnb2a*, *ephb4a*, and *ephb4b* mutants.

**a**, Generation of the *efnb2a* and *efnb2b* mutants using CRISPR/Cas9 technology with a target site at exon 4 and exon 2. The *efnb2a*^-/-^ mutant bears a 7-bp deletion with a protein length of 182 aa. The amino acid sequence changes after 168 aa, preserving the receptor binding domain (RBD). The *efnb2b*^-/-^ mutant bears a 15-bp deletion and an 8-bp insertion with a protein length of 154 aa. The amino acid sequence changes after 125 aa. **b**, Generation of the *ephb4a* mutant using CRISPR/Cas9 technology with a target site at exon 3. The *ephb4a*^-/-^ mutant bears a 1-bp deletion and 11-bp insertion with a protein length of 61 aa. The amino acid sequence changes after 57 aa. The *ephb4a*^-/-^ mutant protein only has a partial ligand binding domain (LBD). **c**, *efnb2a*^-/-^ mutants exhibit abnormal blood vessel formation and blood accumulation in the tail region at 2 dpf. Scale bar, 200 μm. **d**, Percentage of *efnb2a*^-/-^ and *efnb2b*^-/-^ mutants with the pericardial edema phenotype at 4 dpf. Unpaired two-tailed *t* test (WT n = 7; *efnb2a*^-/-^ n = 4; *efnb2b*^-/-^ n = 7). Each dot represents the percentage of edema embryos (total embryos > 40) from one pair of fish with the indicated genotype. **e**, Appropriate development of facial lymphatic vessels labeled by *lyve1b:TopazYFP* in *efnb2a^-/-^;efnb2b^-/-^* double mutants. n, the number of embryos observed. Scale bars, 50 μm. **f**, *ephb4a*^-/-^ mutants show aberrant blood vessel formation and blood accumulation in the tail region at 2 dpf. Scale bars, 200 μm. **g**, *ephb4b*^-/-^ mutants exhibit normal blood circulation at 3 dpf. Scale bar, 500 μm. **h**, Appropriate development of trunk blood vessels in *ephb4b^-/-^* mutants. *flk:mCherry* labels blood vessels and *lyve1b:TopazYFP* labels the notochord, veins and lymphatics. Scale bars, 50 μm. **i**, A typical *ephb4b*^-/-^ adult fish on the Tubingen background shows a hemorrhage-like phenotype with edema in the heart and abdomen (arrowheads) and protrusion of scales (arrows). Scale bars, 1 mm. **j**, Quantification of the hemorrhage-like phenotype in *ephb4b*^-/-^ mutants along with embryonic development in (i). n = the number of fish analyzed. **k**, Uncropped images presented in Fig. 6e, showing *gata1:DsRed* labeled red blood cells (arrows) enter the *lyve1b:TopazYFP* labeled facial lymphatic vessels (green) at 77 hpf in *ephb4b*^-/-^ mutants. Scale bars, 50 μm. **l**, Top, in juvenile *ephb4b*^-/-^ mutants, the blood accumulates in *lyve1b:TopazYFP*-labeled lymphatic vessels (green). Bottom, *gata1:DsRed*-expressing red blood cells accumulate in lymphatic vessels with *lyve1b:TopazYFP* expression in *ephb4b^-/-^* larvae at 1 mpf. Scale bars, 200 μm.


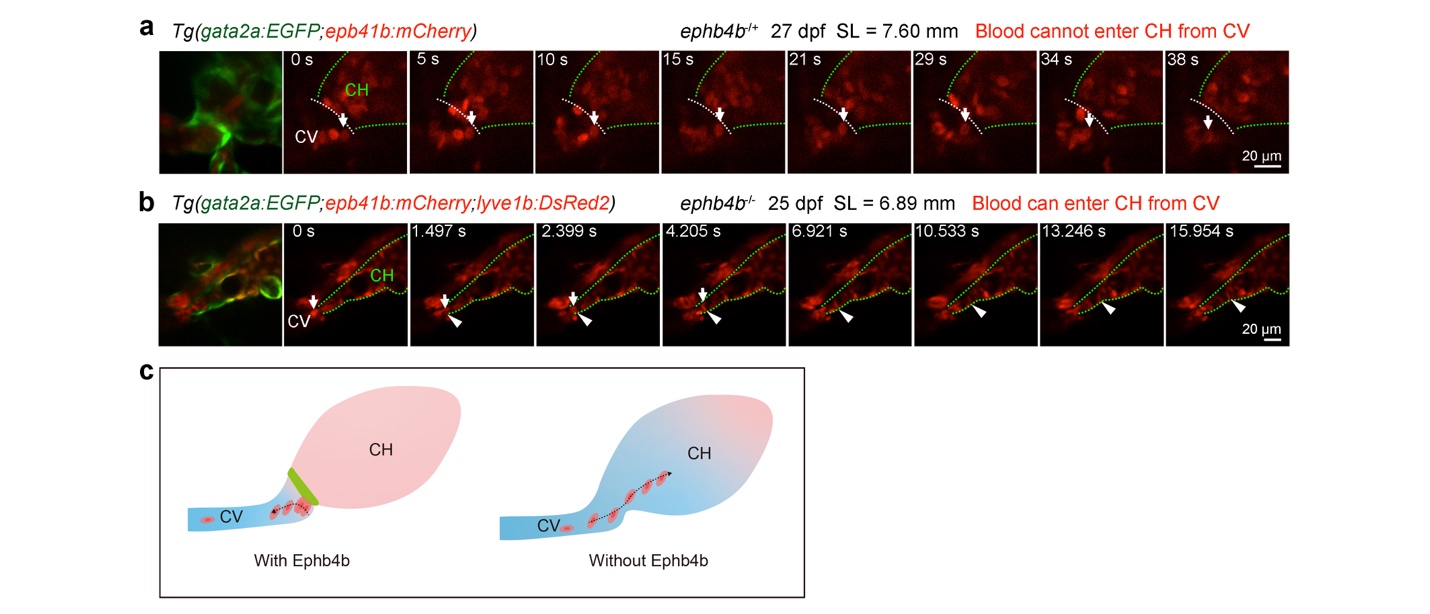


Figure. S10. Defective valves in *ephb4b^-/-^* caudal heart allowed blood to flow from the caudal vein into the caudal heart.

**a**, CH-CV LVV blocks red blood cells (RBCs; red, *Tg(epb41b:mCherry)*) entry of the caudal heart (CH). White lines, CH-CV LVV; green lines, CH. Arrows indicate the flow of one RBC. CV, caudal vein. Scale bar, 20 μm. **b**. The absence of CH-CV LVV in *ephb4b*^-/-^ mutants allows RBCs to enter the CH (green lines). Arrows and arrowheads indicate the flow of two individual RBCs in the caudal heart. Scale bar, 20 μm. **c**, Diagrams of blood flow with or without the CH-CV LVV. All images are anterior to the left, dorsal upward.


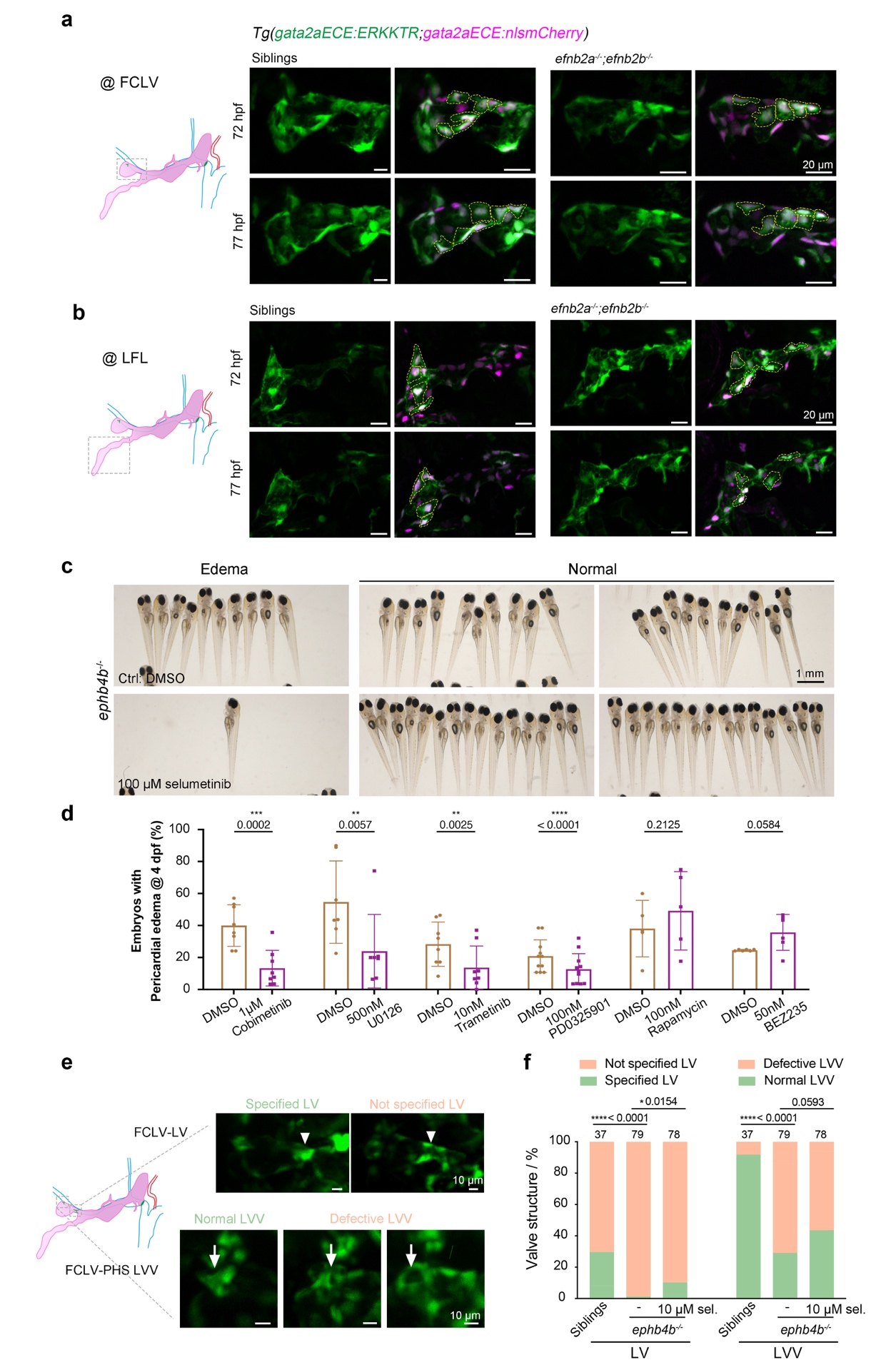


Figure. S11. Activated Erk activity in *efnb2^-/-^* mutants and rescue of pericardial edema and valve formation in *ephb4b^-/-^* mutants.

**a**-**b**, Erk activity detected by ERK-KTR biosensor in *Tg(gata2aECE:ERKKTR;gata2aECE:nlsmCherry)* fish at the FCLV and aLFL in *efnb2a^-/-^;efnb2b^-/-^* double mutants at 72 hpf and 77 hpf. The individual cells used for ERK-KTR-EGFP analysis are indicated by yellow dashed lines. All images are anterior to the left, dorsal upward. Scale bars, 20 μm. Related to Fig. 7c. **c**, Representative results of the treatment of *ephb4b*^-/-^ mutant larvae with DMSO or 10 μM selumetinib. Related to Fig. 7e. Scale bar, 1 mm. **d**, MEK inhibitors cobimetinib, U0126, trametinib, and PD0325901, but not mTOR inhibitors rapamycin and BEZ235, can restore the pericardial edema defects in *ephb4b*^-/-^ mutants at 4 dpf. Paired two-tailed *t* test (At least 3 independent experiments; cobimetinib n = 8; U0126 n = 7; trametinib n = 8; PD0325901 n = 11; rapamycin n = 5; BEZ235 n = 6). **e**, Classification of the defective LV and FCLV-PHS LVV structures in *ephb4b*^-/-^ mutants. In mutants at 77 hpf, both LVs (arrowheads) and FCLV-PHS LVVs (arrows) can be divided into two categories depending on their morphological integrity, including specified and not specified LV, and normal and defective LVV, respectively. Scale bars, 10 μm. **f**, Statistical summary of the selumetinib rescue effects on LV and LVV structures in *ephb4b*^-/-^ mutants at 77 hpf in (e). Chi-squared test, and the sum of three independent experiments.

Captions for movies

Movie S1.

**3D movie of the RFLS structure in *Tg(lyve1b:TopazYFP;flk:mCherry)* background at 30 dpf.** Related to Figure 1a.

**Movie S2**.

**The RFLS structure connects with both facial lymphatic vessels and trunk lymphatic vessels in *Tg(lyve1b:DsRed2)* background.** Lateral lymph., superficial lateral lymphatic vessels. Related to Figure 1a.

Movie S3.

**Lymphovenous valve movement.** FCLV-PHS LVV (white arrows) and RFLS-CCV LVV (yellow arrows) movements at 3.5 dpf. Related to Figure 1b and 1c.

Movie S4.

**Blood flow in the caudal heart and red blood cells entered tail lymphatics.** The caudal heart (yellow arrow) was filled with blood cells that traveled from the caudal fin into the caudal vein. Following that, a 3D view of caudal heart and red blood cells in *Tg(lyve1b:TopazYFP;epb41b:mCherry)* fish at 15 dpf was shown. Individual red blood cells were observed flowing from the tail into the tail lymphatic vessels.

**Movie S5.**

**Z-stack movie of the caudal heart labeled by *fli1a:EGFP*.** Arrow indicates the direct connection of caudal heart to caudal vein. Related to Figure 1f.

**Movie S6**.

**3D movie of the caudal heart in *Tg(lyve1b:TopazYFP;flk:mCherry;gata1:DsRed)* background.** Related to Figure 1g.

**Movie S7**.

The lymphangiography shows a direct link between the CH and CV. 2,000 kDa Dextran-Rhodamine was injected into the tail lymphatics.

Movie S8.

**Structure of the caudal heart in the tail.** The caudal heart and its surrounding lymphatic vessels in larvae fish at 26 dpf. Related to Figure 1h.

**Movie S9**.

**Z-stack movie of the caudal heart labeled by *gata2a:EGFP;lyve1b:DsRed2*.** Related to Figure 1h.

Movie S10.

**3D movie of Erk reporter fish *Tg(gata2aECE:ERKKTR;gata2aECE:nls-mCherry)* at 54 hpf and 78 hpf.** ERKKTR could be observed in a subpopulation of LECs.

Movie S11.

**Time-lapse movie of Erk reporter fish *Tg(gata2aECE:ERKKTR;gata2aECE:nls-mCherry)* from 72 to 78 hpf.** Individual cells in FCLV with increased nuclear ERKKTR fluorescence were marked by white arrows and an enlargement of these cells was shown at top right. Yellow arrow indicated a cell in aLFL with little change of nuclear ERKKTR fluorescence.

Movie S12.

**Lack of lymphatic valve in *efnb2a^-/-^;efnb2b^-/-^* at 77 hpf.** Prox1a immunostaining reveals the absence of high-Prox1a-expressing valve cells in *efnb2a^-/-^;efnb2b^-/-^* mutant. Related to Figure 6h.

Movie S13.

**FCLV-PHS LVV can block RBC flow from PHS into facial lymphatic vessels.** In *ephb4b*^-/-^ mutants, the absence of FCLV-PHS LVV allows RBCs (red, *gata1:DsRed*) to enter the facial lymphatic vessels. Related to Figure 6m.

Movie S14.

**CH-CV LVV can block RBC flow from caudal vein into caudal heart.** In *ephb4b*^-/-^ mutants, the absence of CH-CV LVV allows RBCs to enter the caudal heart. Related to Supplementary Figure 10.

Table S1.

Abbreviation table

| Abbreviation | Full name |
| --- | --- |
| CA | Caudal artery |
| CCV | Common cardinal vein |
| CCLA | Central conducting lymphatic anomaly |
| CH | Caudal heart |
| CM-AVM | Capillary malformation-arteriovenous malformation |
| CV | Caudal vein |
| DA | Dorsal aorta |
| Erk | Extracellular-signal-regulated kinas |
| FCLV | Facial collecting lymphatic vessel |
| FLS | Facial lymphatic sprouts |
| FLV | Facial lymphatic vessel |
| GAP | GTPase activating protein |
| LEC | Lymphatic endothelial cell |
| LFL | Lateral facial lymphatic vessel |
| LL | Superficial lateral lymphatic vessel |
| LV | Lymphatic valve |
| LVV | Lymphovenous valve |
| MAPK | Mitogen-activated protein kinase |
| OLV | Otolithic lymphatic vessel |
| PCV | Posterior cardinal vein |
| PHS | Primary head sinus |
| Rasa1 | Ras p21 protein activator 1M |
| RFLS | Remaining facial lymphatic sprouts |
| TD | Thoracic duct |
| TEM | Transmission electron microscopy |
| VOGM | Vein of Galen malformation |

Table S2.

Zebrafish mutant lines used in this study

| Gene | Allele | Mutagen | Target | Lesion | Allele characteristic |
| --- | --- | --- | --- | --- | --- |
| *ephb4b* | *tsu25* | Cas9 | (J. Zhang et al., 2016)^1^ | 25 bp deletion | Frameshift after aa 106; additional 48 aa then stop |
| *ephb4a* | *tsu37* | Cas9 | GGAGGTCAGTGGTCTGGATGagg | 1 bp deletion and 11 bp insertion | Frameshift after aa 57; additional 4 aa then stop |
| *efnb2a* | *tsu41* | Cas9 | GGGTAGTCTTTGGGGGAAATggg | 7 bp deletion | Frameshift after aa 168; additional 14 aa then stop |
| *efnb2b* | *tsu42* | Cas9 | GGGGCCTGGAGTTCTTAAGAggg | 15 bp deletion and 8 bp insertion | Frameshift after aa 125; additional 29 aa then stop |
| *rasa1a* | *tsu38* | Cas9 | GGCGGTCGCTCTCTCTGATGagg | 2 bp deletion and 18 bp insertion | Frameshift after aa 151; additional 65 aa then stop |
|  | *tsu40* | Cas9 | GGCGGTCGCTCTCTCTGATGagg | 3 bp deletion | 152I deletion |
| *rasa1b* | *tsu39* | Cas9 | GGGATACAGATCTCAGACCTggg  GGGGGATACTCTGGATGTGCagg | 308 bp deletion and 31 bp insertion | Frameshift after aa 40; additional 21 aa then stop |
| *erk1* | *tsu45* | Cas9 | ﻿GGCTCGAATAGCAGCGCCGCcgg | 47 bp deletion | Frameshift after aa 19; additional 15 aa then stop |
| *erk2* | *tsu46* | Cas9 | ﻿GGCGGCCCGAACCCCGGATCcgg | 5 bp deletion | Frameshift after aa 15; additional 35 aa then stop |

Table S3.

Oligonucleotides used in this study

| Name | Sequence | Notes |
| --- | --- | --- |
| ephb4a_Cas9 | taatacgactcactataGGAGGTCAGTGGTCTGGATGgttttagagctagaa | Oligos for sgRNA template amplification |
| efnb2a_Cas9 | taatacgactcactataGGGTAGTCTTTGGGGGAAATgttttagagctagaa |  |
| efnb2b_Cas9 | taatacgactcactataGGGGCCTGGAGTTCTTAAGAgttttagagctagaa |  |
| rasa1a_E2_Cas9 | taatacgactcactataGGCGGTCGCTCTCTCTGATGgttttagagctagaa |  |
| rasa1b_E1_Cas9_1 | taatacgactcactataGGGATACAGATCTCAGACCTgttttagagctagaa |  |
| rasa1b_E1_Cas9_2 | taatacgactcactataGGGGGATACTCTGGATGTGCgttttagagctagaa |  |
| erk1_E1_Cas9 | taatacgactcactataGGCTCGAATAGCAGCGCCGCgttttagagctagaa |  |
| erk2_E1_Cas9 | taatacgactcactataGGCGGCCCGAACCCCGGATCgttttagagctagaa |  |
| gRNA_rev primer (tracr_rev) | AAAAAAAGCACCGACTCGGTGCCAC |  |
| ephb4b^tsu25^_F  ephb4b^tsu25^_R | TTTATCCCCCGCCACGGC  TAGTGTTACAGCTGGCAAGG | 4% agarose gel; mutants appear as a single, smaller band. |
| ephb4a^tsu37^_F  ephb4a^tsu37^_R | GTCGCAGCTTCTGGACTTT  GTCCGCCTGGCAGATCTGA | 4% agarose gel; mutants appear as a single, larger band. |
| efnb2a^tsu41^_wtF  efnb2a^tsu41^_R | AGACCCCTCTGATCCCATT  CCTTGCCCCCTAAGTCAG | Mutant cannot be amplified by wtF + R primer. |
| efnb2a^tsu41^_F2  efnb2a^tsu41^_R2 | CTGTGTTTTGCAGACCCC  CCTTGCCCCCTAAGTCAG | 4% agarose gel; heterozygotes have 2-3 bands. |
| efnb2b^tsu42^_F  efnb2b^tsu42^_R | TGTCCCAGGAAGAGGTGTA  TAGTGTTACAGCTGGCAAGG | Wild-type can be cut by *Afl*II. |
| rasa1a^tsu38^_F  rasa1a^tsu38^_R | AGATGGTACCATGGAATGTT  TCATGATAGTAAAGGCCACTC | 4% agarose gel; mutants have a single, larger band. |
| rasa1a^tsu40^_F  rasa1a^tsu40^_R | AGATGGTACCATGGAATGTT  TCATGATAGTAAAGGCCACTC | *Dde*I; wild-type has one cut site, mutant has two cut sites; 4% agarose gel. |
| rasa1b^tsu39^_F  rasa1b^tsu39^_F | ATGATGGCAACCCAGGGTGG  CCAAAGCATCACTCACTGAT | 4% agarose gel; mutants have a single, smaller band. |
| erk1^tsu45^_F  erk1^tsu45^_R | ﻿GATCTGAAAGCAAATACGAGGC  ﻿TACTGGAGATCAGTGTATCGGG | 4% agarose gel; mutants have a single, smaller band. |
| erk2^tsu46^_F  erk2^tsu46^_R | ﻿ACCGAGTCTTCGGTTCAGTTTA  ﻿GTAGGAGAGGTTGCTGTAACGC | Wild-type can be cut by *Bam*HI; 4% agarose gel. |
| erk1_F  erk1_T7_R | gtggctactcgctggtacc  taatacgactcactataggggcccaaactagcgcacagc | Primers for probe synthesis |
| lyve1b_promoter_F | TGATATTACGGATGCCCTCTCT | Primers for transgenic plasmid construction |
| lyve1b_promoter_R | TTGGATTTCACCCACGTC |  |
| epb41b_promoter_F | ACCTCGCAATACTGCTGCTG |  |
| epb41b_promoter_R | GTTTCTCAAACCTTTGTCTT |  |

Reference

1 Zhang, J., Jiang, Z., Liu, X. & Meng, A. Eph/ephrin signaling maintains the boundary of dorsal forerunner cell cluster during morphogenesis of the zebrafish embryonic left-right organizer. *Development*. **143**, 2603-2615, (2016).
